# Regulated Release of Cryptococcal Polysaccharide Drives Virulence and Suppresses Immune Cell Infiltration into the Central Nervous System

**DOI:** 10.1128/IAI.00662-17

**Published:** 2018-02-20

**Authors:** Steven T. Denham, Surbhi Verma, Raymond C. Reynolds, Colleen L. Worne, Joshua M. Daugherty, Thomas E. Lane, Jessica C. S. Brown

**Affiliations:** a Division of Microbiology and Immunology, Pathology Department, University of Utah School of Medicine, Salt Lake City, Utah, USA; University of Cincinnati

**Keywords:** Cryptococcus neoformans, capsule, glucuronoxylomannan, host-pathogen interaction, immune suppression, virulence

## Abstract

Cryptococcus neoformans is a common environmental yeast and opportunistic pathogen responsible for 15% of AIDS-related deaths worldwide. Mortality primarily results from meningoencephalitis, which occurs when fungal cells disseminate to the brain from the initial pulmonary infection site. A key C. neoformans virulence trait is the polysaccharide capsule. Capsule shields C. neoformans from immune-mediated recognition and destruction. The main capsule component, glucuronoxylomannan (GXM), is found both attached to the cell surface and free in the extracellular space (as exo-GXM). Exo-GXM accumulates in patient serum and cerebrospinal fluid at microgram/milliliter concentrations, has well-documented immunosuppressive properties, and correlates with poor patient outcomes. However, it is poorly understood whether exo-GXM release is regulated or the result of shedding during normal capsule turnover. We demonstrate that exo-GXM release is regulated by environmental cues and inversely correlates with surface capsule levels. We identified genes specifically involved in exo-GXM release that do not alter surface capsule thickness. The first mutant, the *liv7*Δ strain, released less GXM than wild-type cells when capsule was not induced. The second mutant, the *cnag_00658*Δ strain, released more exo-GXM under capsule-inducing conditions. Exo-GXM release observed *in vitro* correlated with polystyrene adherence, virulence, and fungal burden during murine infection. Additionally, we found that exo-GXM reduced cell size and capsule thickness under capsule-inducing conditions, potentially influencing dissemination. Finally, we demonstrated that exo-GXM prevents immune cell infiltration into the brain during disseminated infection and highly inflammatory intracranial infection. Our data suggest that exo-GXM performs a distinct role from capsule GXM during infection, altering cell size and suppressing inflammation.

## INTRODUCTION

Cryptococcus neoformans is a globally distributed saprophytic fungus found associated with certain species of trees and bird droppings (1). Due to the global environmental distribution of C. neoformans, human exposure to C. neoformans is almost universal (1, 2). Exposure occurs via inhaled fungal spores or desiccated yeast cells that enter the lungs, where they are either cleared by the immune system or contained in a persistent state for a decade or more (3). However, in immunocompromised hosts C. neoformans cells can disseminate from the lungs to basically any organ in the body (4). C. neoformans proliferates particularly well in the brain, resulting in life-threatening meningoencephalitis (5). Cryptococcal infections are responsible for 15% of AIDS-related deaths worldwide, with meningoencephalitis being the primary cause of death (6). Most cases occur in sub-Saharan Africa and Asia, with mortality rates exceeding 50% in resource-poor areas (6).

In contrast to many forms of bacterial and viral meningitis, cryptococcal meningoencephalitis is associated with strikingly low levels of inflammation and infiltrating immune cells into the central nervous system (CNS) of both human patients and mouse models (7–11). This paucity of inflammation is linked to poorer clinical outcomes and subdued clinical signs that can delay treatment (9, 12, 13).

An essential factor for C. neoformans virulence is the conditional production of a thick polysaccharide surface capsule, which can more than double the diameter of a C. neoformans cell (14). The primary capsule constituent is glucuronoxylomannan (GXM), which comprises approximately 90% of the capsule mass (15, 16). Surface capsule plays a number of different roles during pathogenesis, protecting C. neoformans cells from phagocytosis, complement, and oxidative stress (15, 17, 18). GXM also has numerous immunomodulatory properties that facilitate fungal survival in the host (19). Notably, GXM increases anti-inflammatory cytokine (interleukin-10 [IL-10]) release while dampening proinflammatory cytokine release (IL-12, gamma interferon [IFN-γ], tumor necrosis factor alpha [TNF-α], IL-1B, and IL-6) (20–23). GXM disrupts antigen presentation by macrophages and dendritic cells and can even induce macrophage apoptosis, thereby diminishing T cell proliferation (21, 24–26). GXM can also suppress leukocyte infiltration into sites of inflammation (27–29).

GXM noncovalently attaches to the cell surface during cell surface capsule formation and maintenance (16). However, it is also found free within the extracellular milieu. This exo-cellular GXM (exo-GXM) reaches milligram/milliliter concentrations in laboratory growth medium (30) and can be observed in the high-microgram/milliliter range in patient serum and cerebrospinal fluid (10, 31). GXM serum titers in HIV-associated cryptococcosis patients positively correlate with nonprotective immune signatures and increased mortality (32).

Despite longstanding knowledge of the existence of exo-GXM, its connection to cell-associated GXM and the mechanisms behind its release remain largely unclear. One hypothesis is that exo-GXM is shed mechanically from the cell surface capsule (16, 33). Alternatively, it has been speculated that distinct mechanisms might regulate the production of cell-associated GXM and exo-GXM in response to environmental cues (15, 16, 34). The latter hypothesis is supported by observations that cell-associated GXM and exo-GXM display different biophysical properties (34). Decreased electromobility of exo-GXM under capsule-inducing conditions indicates that these differences could occur at the level of polymer length or branching (35–37).

Here, we test the hypothesis that exo-GXM production is regulated by environmental conditions. We find that exo-GXM production is inversely related to the thickness of the cell surface-retained capsule and identify genes involved in these processes. Exo-GXM production also correlates with virulence and reduces infiltration of immune cells into the CNS during infection. Together, these data support the idea that exo-GXM plays a critical role that is distinct from that of cell surface GXM during infection.

## RESULTS

### Environmental signals alter exo-GXM levels.

**FIG 1:**
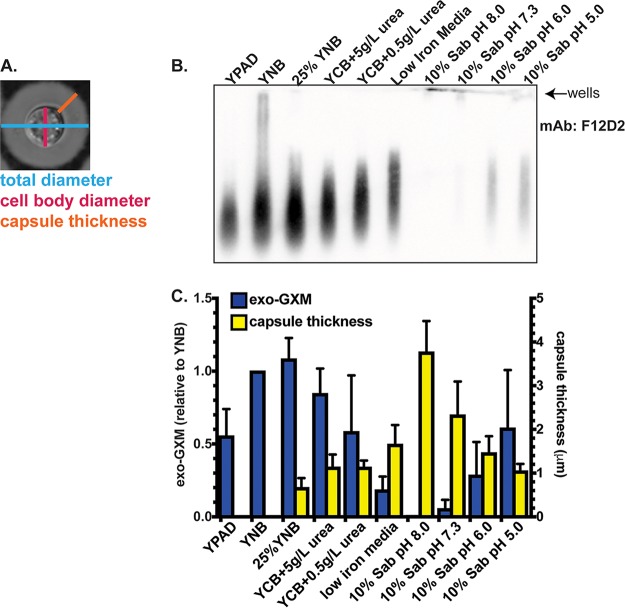
Levels of detectable exo-GXM negatively correlate with capsule thickness under a variety of medium conditions. To generate conditioned medium, we normalized 24-h cultures by cell density and then passed the supernatants through a 0.22-μm-pore-size filter to remove fungal cells. (A) Representative image of cell/capsule measurements used in this study. (B) We tested supernatants for free GXM (exo-GXM) by blotting and probing samples with the F12D2 anti-GXM MAb. See Materials and Methods for further details. A representative blot showing relative levels of exo-GXM collected from cells cultured for 24 h under a variety of capsule- and non-capsule-inducing conditions is shown. (C) The intensities of exo-GXM bands relative to those of exo-GXM in YNB–2% glucose cultures (blue bars) were quantitated for three independent experiments and plotted next to absolute measurements of capsule thickness (yellow bars) (*n* = 30 cells). Data were combined from three independent experiments. Bars represent the means, and error bars represent the standard deviations. Sab, Sabouraud's medium.

To investigate whether exo-GXM release results from passive shedding of surface capsule or is regulated at some level, we cultured wild-type C. neoformans cells for 24 h under a variety of medium conditions. We then measured capsule size and the amount of exo-GXM released into the medium. We chose both non-capsule-inducing medium and a series of capsule-inducing media intended to produce a range of levels of capsule induction. We harvested cells and then stained them with india ink to measure capsule thickness as the distance from the cell wall to the outer capsule edge (Fig. 1A). We filtered supernatant through a through a 0.22-μm-pore-size filter to remove cells, and then immunoblotted samples with the anti-GXM monoclonal antibody (MAb) F12D2 to quantify exo-GXM release as relative staining intensity (Fig. 1B). Exo-GXM band intensities were normalized to the level of yeast nitrogen base (YNB) plus 2% glucose, which was the condition with the greatest observed levels of exo-GXM.

We found an inverse relationship between capsule thickness and exo-GXM, such that cells growing under the strongest capsule-inducing conditions, like 10% Sabouraud's medium buffered to alkaline pH, produced the least amount of exo-GXM (Fig. 1B and C). This relationship held across other capsule-inducing conditions, such as nitrogen and iron limitation, that produce intermediate levels of both cell surface GXM and exo-GXM.

GXM is an α-1,3-mannan backbone with branching glucuronic acid and xylose residues and variable 6-*O*-acetylation on the backbone (38). *O*-Acetylation varies across strains and is not required for capsule formation but significantly affects GXM's immunoreactive properties (38–40). Deletion of *CAS1*, which is required for *O*-acetylation, results in a hypervirulent phenotype (41). We analyzed the same conditioned medium as used in the experiment shown in Fig. 1 but used the MAb 1326 to detect GXM. MAb 1326 recognizes *O*-acetylated GXM but is unable to recognize non-*O*-acetylated GXM. F12D2, on the other hand, recognizes both *O*-acetylated and non-*O*-acetylated GXM. Thus, 1326 staining intensity relative to F12D2 intensity reflects the relative proportion of *O*-acetylated GXM present in the supernatant. We observed that 1326 staining relative to F12D2 staining increased under certain capsule-inducing conditions (low nitrogen, low iron, and 10% Sabouraud's medium, pH 5 to 6), indicative of proportionally increased *O*-acetylated GXM (see Fig. S1 in the supplemental material). These results demonstrate that environmental conditions may also influence GXM modification, specifically *O*-acetylation, with potential implications for immune recognition.

### Identification of gene deletion mutants with reduced exo-GXM secretion under non-capsule-inducing conditions.

We then identified mutants with reduced GXM production. We screened the C. neoformans partial knockout collection (CM18 background, 1,200 targeted gene knockouts) (42) under YNB conditions, which results in high exo-GXM production. We grew each strain for 24 h at 37°C, removed the cells by centrifugation, and then probed the conditioned medium for exo-GXM.

**FIG 2:**
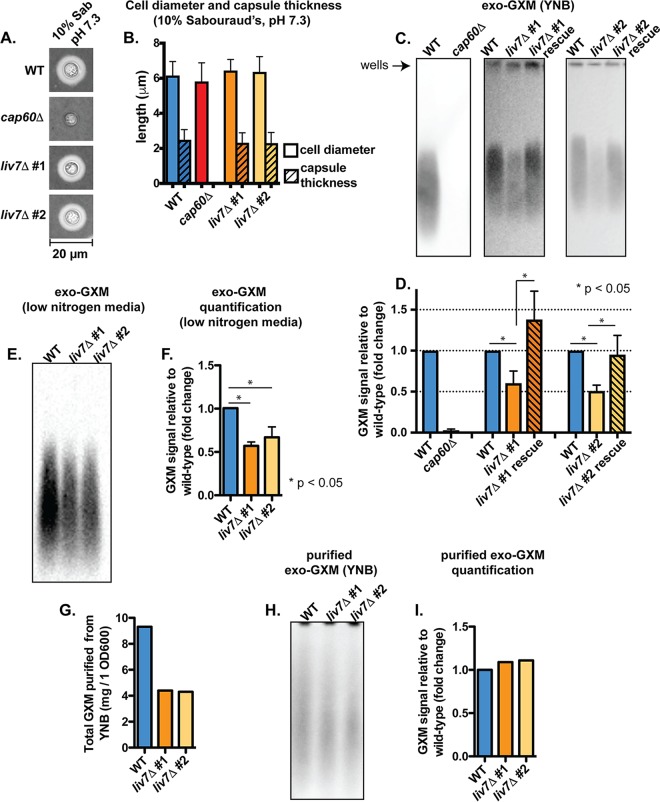
Identification of a genetic mutant (*liv7*Δ strain) with reduced exo-GXM release but no observable changes to capsule thickness. (A) Representative india ink images of cells grown in 10% Sabouraud's dextrose medium, pH 7.3, for 24 h. Capsule thicknesses were similar across KN99 wild-type (WT) cells and cells from each independent *liv7* deletion mutant (*liv7*Δ 1 and *liv7*Δ 2). (B) Quantification of cell body diameter and capsule thickness from three independent experiments (*n* = 30 cells per strain). Bars represent means with standard deviations. (C) Conditioned media from cultures of wild type, *liv7*Δ mutants 1 and 2, and *liv7*Δ mutants 1 and 2 with the *liv7* gene reintroduced into its native locus (*liv7*Δ rescue) grown under non-capsule-inducing conditions (YNB plus 2% glucose) for 24 h. Blots were probed with anti-GXM antibody F12D2. (D) Quantification of blot signal intensities shows reduced exo-GXM release by *liv7*Δ mutant 1/2 cells. Data were combined from three independent experiments. *P* values were calculated using a Mann-Whitney test; bars represent means with standard deviations. (E) Conditioned medium from wild-type and mutant cultures grown under mild capsule-inducing conditions (YCB with 0.5g/liter urea) for 24 h. Blots were probed with anti-GXM antibody F12D2. (F) Quantification of blot signal intensities shows reduced exo-GXM release by *liv7*Δ mutant 1/2 cells under mild capsule-inducing conditions. Data were combined from three independent experiments. *P* values were calculated using a Mann-Whitney test; bars represent means with standard deviations. (G) Twofold less GXM, by mass, was purified from *liv7*Δ mutant 1/2 cells than from wild-type cells grown for 72 h in YNB–2% glucose. (H) Purified GXM from wild-type and *liv7*Δ mutant 1/2 cells was resuspended at a concentration of 1 mg/ml and immunoblotted with MAb F12D2. (I) Quantification of signal intensities from the blot in shown in panel H shows similar intensities for GXM purified from wild-type and *liv7*Δ mutant 1/2 cells.

We searched the YNB-grown mutants for samples that produced less exo-GXM than wild-type cells. We then stained induced cell surface capsule (by growth in 10% Sabouraud's medium, pH 7.3) in this subset of mutants and eliminated any with a growth defect and/or a substantial (>25%) reduction in cell surface capsule thickness. We also stained for common pathogen-associated molecular patterns (PAMPs), such as exposed mannoproteins and chitin, which activate host immune responses (43). This left us with a single mutant, the *cnag_06464*Δ, or *liv7*Δ, strain, which we reconstructed in the KN99 genetic background (Fig. 2). Four other mutants (Table S1) exhibited a moderate defect in cell surface capsule in addition to their moderate defects in exo-GXM release. However, we focused on the *liv7*Δ mutant because of its ability to form wild-type levels of cell surface capsule.

The *LIV7* gene was previously identified in a screen for mutants deficient in competitive growth in the lungs after being pooled with 47 other gene deletion mutants (42). Liv7 is localized to the Golgi complex under capsule-inducing conditions (Dulbecco's modified Eagle's medium [DMEM] plus 5% CO_2_) (44). *liv7*Δ cells produce wild-type-like levels of cell surface capsule when grown in 10% Sabouraud's medium, pH 7.3 (Fig. 2A and B), but conditioned medium from *liv7*Δ cell cultures grown in YNB contains 2-fold less GXM than conditioned medium from wild-type C. neoformans cell cultures (Fig. 2C and D). PAMP exposure is comparable to that of wild-type cells (Fig. S2A and B).

The environment within the host is highly variable, and capsule sizes within different host niches also vary. While capsule is strongly induced in the lungs, capsule size varies considerably between strains and throughout infection (45, 46), including during phagocytosis by macrophages (45). We were interested in whether *liv7*Δ cells also released less exo-GXM under any sort of capsule induction. Indeed, under mild capsule induction in low-nitrogen medium (yeast carbon base [YCB] plus 0.5 g/liter urea), we observed a reduction in exo-GXM release by *liv7*Δ cells (Fig. 1C and 2E and F).

We also took measures to ensure that the relative levels of exo-GXM analyzed by immunoblotting reflected the actual abundance of exo-GXM in culture supernatant rather than a change in GXM's antigenicity. We purified GXM from the supernatant of wild-type and *liv7*Δ cells grown for 72 h in YNB. Not only did we purify half as much exo-GXM from *liv7*Δ cells as wild-type cells (Fig. 2G), but also after immunoblotting equal masses of wild-type and *liv7*Δ cell exo-GXM, we did not detect a substantial difference in signal intensities (Fig. 2H and I).

### Identification of gene deletion mutants with elevated exo-GXM secretion under strong capsule-inducing conditions.

**FIG 3:**
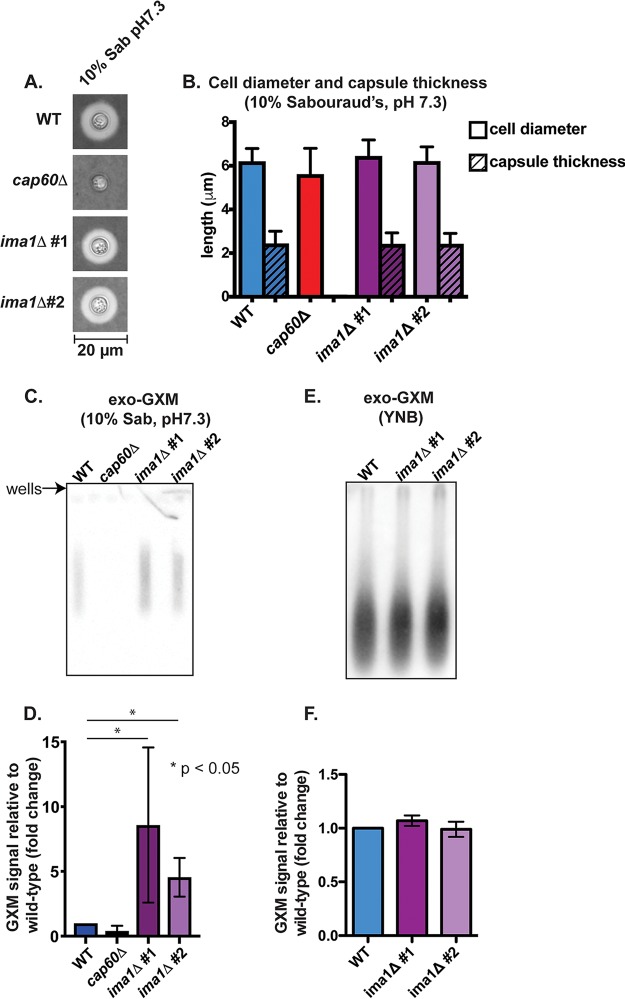
Identification of a genetic mutant (*ima1*Δ strain) with increased exo-GXM release but no observable changes to capsule thickness. (A) Representative india ink images of cells grown in 10% Sabouraud's dextrose medium, pH 7.3, for 24 h. Capsule thicknesses were similar across KN99 wild-type (WT) cells and cells from each independent *ima1* mutant (also *cnag_00658*; see main text for details) deletion strain (*ima1*Δ #1 and *ima1*Δ #2). (B) Quantification of cell body diameter and capsule thickness from three independent experiments (*n* = 30 cells per strain; bars represent means with standard deviations). (C) Conditioned medium from cultures grown for 24 h under strong capsule-inducing conditions (10% Sabouraud's medium, pH 7.3). Blots were probed with anti-GXM antibody F12D2. (D) Quantification of blot signal intensities shows increased exo-GXM release by *ima1*Δ mutants 1/2 (combined data from three independent experiments). *P* values were calculated using a Mann-Whitney test; bars represent means with standard deviations. (E) Conditioned medium from cultures grown for 24 h under non-capsule-inducing conditions (YNB medium plus 2% glucose). Blots were probed with anti-GXM antibody F12D2. (F) Quantification of blot signal intensities shows similar levels of exo-GXM release by *ima1*Δ mutants 1/2 and wild-type cells (combined data from three independent experiments). *P* values were calculated using a Mann-Whitney test; bars represent means with standard deviations.

We next identified mutants that produced elevated levels of exo-GXM under capsule-inducing conditions when exo-GXM production is very low. We again screened the C. neoformans knockout mutant collection (CM18 background); mutants were grown in YNB and then subcultured at a dilution of 1/100 in 10% Sabouraud's medium, pH 7.3, and grown for 48 h at 37°C. We again removed mutants that exhibited growth defects, elevated PAMP exposure, and a substantial (>25%) reduction in cell surface capsule thickness. We found two groups of mutants: group 1 exhibited approximately wild-type capsule thickness, while group 2 mutants had less than wild-type levels of cell surface capsule (Table S1). We focused our subsequent experiments on the mutant in gene *cnag_00658*, which produces cell surface capsule with the same thickness as wild-type cells (Fig. 3A and B). The *CNAG_00658* gene was previously screened for competitive growth in the lungs as part of a pooled infection with 47 other mutant strains but did not display a significant competitive advantage or disadvantage (42). As with the *liv7*Δ strain, we reconstructed this mutant in the KN99 genetic background and used these strains for all subsequent experiments. As in the CM18 background, *cnag_00658*Δ cells in the KN99 background released increased exo-GXM in 10% Sabouraud's medium, pH 7.3 (Fig. 3C and D). Under non-capsule-inducing conditions (YNB plus 2% glucose), *cnag_00658*Δ cells released levels of exo-GXM equivalent to those of the wild type (Fig. 3E and F). Unlike other mutants in group 1, *cnag_00658*Δ cells produced the same levels of melanin and urease as wild-type cells (Fig. S2C).

We examined whether the observed immunoblot intensities for GXM produced by *cnag_00658*Δ cells were truly due to differences in total abundance and not to any potential effects that the *CNAG_00658* gene might have on GXM's antigenicity. We grew wild-type and *cnag_00658*Δ cells for 72 h in 10% Sabouraud's medium, pH 7.3, but were not able to successfully purify enough GXM from our 50-ml cultures for immunoblotting, perhaps because little exo-GXM is released under strong capsule-inducing conditions, such as 10% Sabouraud's medium, pH 7.3 (Fig. 1C and 3C).

The *CNAG_00658* gene encodes a predicted protein that is 624 amino acids (aa) in length. It shares N-terminal sequence homology with the Schizosaccharomyces pombe inner nuclear membrane protein, IMA1 (615 aa). The predicted product of the *CNAG_00658* gene also has five putative transmembrane domains that positionally align with the five transmembrane domains of the S. pombe IMA1 protein. For these reasons, we propose to rename the *CNAG_00658* gene *IMA1*.

### Changes in exo-GXM levels alter fungal cell adherence.

We had thus far assayed exo-GXM secretion only during planktonic growth. However, within the natural environment of soil and vegetable matter, C. neoformans can form adherent biofilms (47). Previous work on cryptococcal biofilms has revealed that a significant portion of the extracellular matrix is composed of GXM and that it plays a critical role in adherence (48). Acapsular strains are unable to adhere to surfaces such as polystyrene, and the addition of anti-GXM antibodies to developing wild-type biofilms reduces their adherence (48). We speculated that exo-GXM may be incorporated into the extracellular matrix during sessile growth to provide community-level structure and that our exo-GXM mutants would display various levels of adherence corresponding to their exo-GXM secretion profiles.

To test this, we grew cells at a concentration of 10^6^ cells/100 μl in 96-well polystyrene plates at 37°C. After 48 h, the wells were washed forcefully with phosphate-buffered saline (PBS)–0.1% Tween 20 dispensed from an automated plate washer, resuspended in PBS containing XTT/menadione [where XTT is 2,3-bis(2-methoxy-4-nitro-5-sulfophenyl)-2H-tetrazolium-5-carboxanilide], and left for 5 h at 37°C. XTT is reduced by fungal cells to produce a colorimetric measure of metabolism that is highly correlative with viable cell count (49).

**FIG 4:**
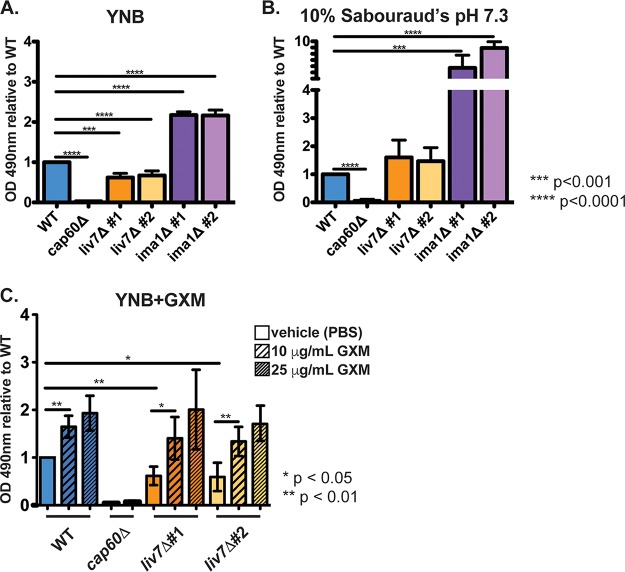
Mutants' alterations to exo-GXM release correlate with adherence. A total of 10^6^ C. neoformans cells was seeded into individual wells of 96-well polystyrene plates and incubated at 37°C. After 48 h, the wells were washed to remove nonadhered and/or weakly adhered cells before resuspension in XTT for colorimetric analysis of metabolic activity as a proxy for viable cell count. (A) OD_490_ readings from cells grown in YNB medium, normalized to wild-type cell readings. *liv7*Δ mutant 1/2 cell adherence was reduced, and *ima1*Δ mutant 1/2 cell adherence was increased compared to that of wild-type cells. (B) OD_490_ readings from cells grown in 10% Sabouraud's medium, pH 7.3, normalized to wild-type cell readings. *ima1*Δ mutant 1/2 cell adherence was increased compared to that of wild-type cells. Combined data from three independent experiments are shown. *P* values were calculated using a Mann-Whitney test; bars represent means with standard deviations. (C) OD_490_ readings from cells grown in YNB medium, normalized to wild-type cell readings. Cells were mixed with purified GXM or vehicle (PBS) prior to inoculation of plates. Wild-type and *liv7*Δ mutant 1/2 cell adherence increased in the presence of GXM.

We constructed two independent knockout mutants, designated mutants 1 and 2, for the genes of interest, *liv7*Δ and *ima1*Δ. Cells of the wild type, *cap60*Δ mutant, *liv7*Δ mutants 1 and 2 (*liv7*Δ mutants 1/2), and *ima1*Δ mutants 1 and 2 (*ima1*Δ mutants 1/2) were assayed in both YNB and 10% Sabouraud's medium at pH 7.3 to replicate planktonic non-capsule- and capsule-inducing conditions, respectively. The *cap60*Δ cells served as a negative control as acapsular mutants are unable to adhere, likely due to their lack of surface and exo-GXM (48). We hypothesized that *liv7*Δ mutant 1/2 cells would display reduced adherence in our assay due to the reduction in exo-GXM release we observed during planktonic growth. This was indeed the case as we observed an approximately 2-fold reduction in the ability of *liv7*Δ mutant 1/2 cells to adhere in our assay (Fig. 4A).

In contrast to growth in YNB, *liv7*Δ mutant 1/2 cells were able to adhere at wild-type levels when grown in 10% Sabouraud's medium (pH 7.3), perhaps because our observations of planktonic cells indicated that far less exo-GXM is released by both wild-type and *liv7*Δ mutant 1/2 cells under these conditions (Fig. 4B). Similarly, *ima1*Δ mutant 1/2 cells, which displayed elevated exo-GXM release under strong capsule-inducing conditions, demonstrated 6- to 8-fold-higher adherence than the wild type when grown in 10% Sabouraud's medium at pH 7.3 (Fig. 4B). When grown in YNB, *ima1*Δ mutant 1/2 cells still displayed increased adherence, but the level was reduced to an approximately 2-fold increase over the wild-type level (Fig. 4A). Moreover, the addition of purified GXM restored *liv7*Δ mutant 1/2 cell adherence back to wild-type levels in a concentration-dependent manner (Fig. 4C). Altogether, these results suggest that the regulated secretion of exo-GXM may have a specialized role in an environmental setting by promoting the adherence of C. neoformans communities.

### Host survival and fungal burden correlate with *in vitro* exo-GXM levels.

Next, we sought to use *liv7*Δ mutants 1/2 and *ima1*Δ mutants 1/2 as an opportunity to explore roles for exo-GXM during pathogenesis. We hypothesized that exo-GXM release would promote virulence through its immunomodulatory properties. Since *liv7*Δ mutant 1/2 and *ima1*Δ mutant 1/2 cells produce wild-type-sized surface capsules in culture, we anticipated that *liv7*Δ mutant 1/2 and *ima1*Δ mutant 1/2 cells would allow us to assess the role of exo-GXM in pathogenesis, independent of surface capsule. We predicted that the reduction in the ability of *liv7*Δ mutant 1/2 cells to produce exo-GXM *in vitro* would result in reduced virulence. Similarly, we predicted that *ima1*Δ mutant 1/2 cells, which show increased exo-GXM under capsule-inducing conditions, would display heightened virulence.

**FIG 5:**
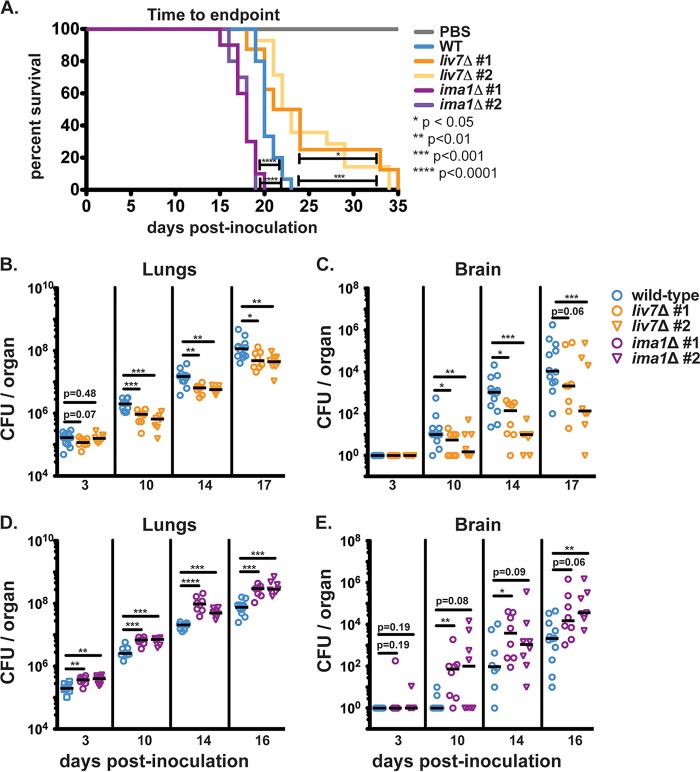
Mutants' alterations to *in vitro* exo-GXM release correlate with changes in survival and fungal burden during infection. (A) C57BL/6NJ mice inoculated intranasally with *ima1*Δ mutant 1/2 (*n* = 10 for both) reach endpoint significantly sooner than wild-type-infected mice (*n* = 15). Wild-type-infected mice reached endpoint sooner than *liv7*Δ mutant 1/2-infected mice (*n* = 8 and *n* = 14, respectively). Mock-infected animals given sterile 1× PBS (*n* = 5) did not show signs of disease 35 days postinoculation. *P* values were calculated using a log rank (Mantel-Cox) test. (B) *liv7*Δ mutant 1/2-infected mice (*n* = 8 for both) show decreased lung burden beginning at 10 days postinoculation compared to the wild-type level (*n* = 12). (C) Brain fungal burden is significantly lower in *liv7*Δ mutant 1/2-infected animals than wild-type-infected animals. (D) Lung fungal burden is significantly higher in *ima1*Δ mutant 1/2-infected mice (*n* = 8 for both) than in wild-type-infected mice (*n* = 8 on days 3, 10, and 14; *n* = 12 on day 16) beginning at least 3 days postinoculation. (E) Dissemination to the brain trends higher in *ima1*Δ mutant 1/2-infected mice. *P* values were calculated using a Mann-Whitney test.

We employed a murine model of disseminated cryptococcosis by inoculating C57BL/6NJ mice (Jackson Laboratory) intranasally with 2.5 × 10^4^ fungal cells per mouse. We calculated survival as the time it took each mouse to reach 85% of its initial mass. Consistent with our hypothesis, *in vitro* exo-GXM production inversely correlated with host survival. Wild-type KN99-infected mice reached endpoint at a median of 20 days postinoculation (dpi). In contrast, *liv7*Δ mutant 1/2-infected mice reached endpoint at a median of 22.5 dpi, and *ima1*Δ mutant 1/2-infected mice reached endpoint at a median of 18 dpi (Fig. 5A). We observed similar survival trends when we inoculated C57BL/6NJ mice with 10-fold-fewer cryptococcal cells (Fig. S3A) or when we inoculated genetically distinct BALB/cJ mice with the exo-GXM mutant strains (Fig. S3B). However, it is important to note that all strains were sufficiently virulent to cause lethal infection. This was not altogether unexpected as the exo-GXM secretion phenotypes for *liv7*Δ mutant 1/2 and *ima1*Δ mutant 1/2 cells were dependent on growth conditions and manifested as a gradient of exo-GXM production rather than as complete ablation or overexpression.

We also assessed fungal burden by plating homogenized organs for CFU counts. Organ fungal burden followed the same trends as survival. Mice inoculated with *liv7*Δ mutant 1/2 cells consistently presented with lower fungal burden in the lungs by day 10 postinoculation (Fig. 5B). Dissemination of *liv7*Δ mutant 1/2 cells to the spleen (Fig. S3D) and brain (Fig. 5C) was also reduced compared to levels of the wild type. In contrast, mice inoculated with *ima1*Δ mutant 1/2 cells suffered higher pulmonary fungal burden than those inoculated with wild-type C. neoformans as early as 3 days postinoculation (Fig. 5D). We also observed a greater number of disseminated *ima1*Δ mutant 1/2 cells in the liver and spleen throughout the course of infection (Fig. S3E and F). *ima1*Δ mutant 1/2 cells disseminated to the brain earlier than wild-type cells, with some *ima1*Δ mutant 1/2 CFU appearing in the brain as early as 3 dpi (Fig. 5E). Total brain fungal burden in *ima1*Δ mutant 1/2-infected mice trended higher than that of the wild type, with one independent gene deletion strain achieving a statistically significant increase in fungal burden at 10 dpi and beyond, despite high variance in dissemination at the observed time points (Fig. 5E). These results suggest that time-to-endpoint for the mice was at least partially due to fungal lung burden and extrapulmonary dissemination, both of which correlated with *in vitro* exo-GXM release.

Since *in vitro* exo-GXM production by *ima1*Δ mutant 1/2 and *liv7*Δ mutant 1/2 cells correlated with virulence *in vivo*, we examined whether or not the *in vitro* phenotypes would translate into detectable differences in exo-GXM production in the host environment. We analyzed the levels of GXM in the lungs, livers, spleens, and brains of infected mice by performing GXM enzyme-linked immunosorbent assays (ELISAs) on filtered (pore size, 0.22 μm) organ homogenates. Exo-GXM levels *in vivo* were highly variable, perhaps reflecting the heterogeneous host environment or assay insensitivity (Fig. S4). In spite of this variability, we detected significant reductions in total exo-GXM in the lungs and extrapulmonary organs of *liv7*Δ mutant 1/2-infected mice at certain time points, with these trends becoming more apparent as infection progressed (Fig. S4A to D). Similarly, *ima1*Δ mutant 1/2-infected mice displayed increased total exo-GXM in the lungs, spleen, and liver by 14 dpi (Fig. S4E to G). We did not observe any interpretable differences in exo-GXM levels on a per-cell basis (data not shown), possibly due to changing host conditions over the course of dissemination (46, 50, 51). For instance, the amount of GXM per CFU fluctuated greatly in the lungs and extrapulmonary organs over the course of infection (Fig. S5A and B). Spread and/or clearance of exo-GXM within the host likely also affects these analyses as the spleen and livers of infected mice had massively increased exo-GXM levels early in infection, when few cryptococcal cells had disseminated to those organs (Fig. S5C).

In addition, we detected exo-GXM in extrapulmonary organs prior to consistent detection of CFU in those same organs (Fig. S5C and D). This observation may be relevant for diagnosticians interested in detecting cryptococcal infection prior to dissemination in at-risk patient populations as early diagnosis of cryptococcosis greatly improves outcomes (52).

### Cell size shifts dramatically during the course of infection in parallel to increases in exo-GXM.

We investigated whether or not *in vitro* capsule phenotypes for the mutants were recapitulated *in vivo*. We isolated cryptococcal cells from infected mice, fixed them with paraformaldehyde, and measured cell body diameter, cell surface capsule thickness, and total diameter (cell diameter including capsule) using india ink (Fig. 1A).

**FIG 6:**
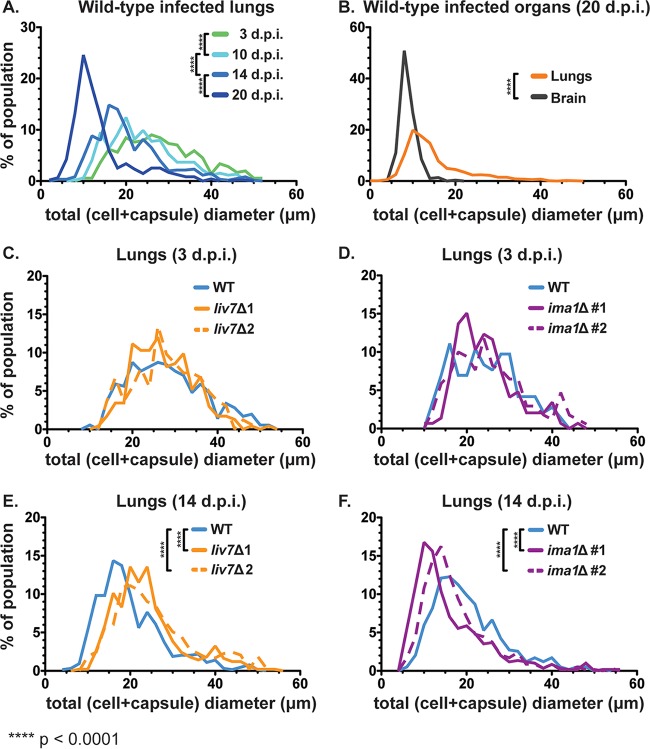
Cell size distributions over the course of infection. We visualized fungal cells from tissue homogenates (from infected mice used in the experiments represented in Fig. 5B to E) in india ink and measured cell size. Total diameter was measured as shown in Fig. 1A. (A) Mean total cell diameter decreases over time within the lungs of wild-type-infected mice as the population shifts toward smaller cells with smaller capsules (*n* = 3 to 4 mice per time point, ≥120 cells per mouse). (B) Disseminated cells found in the brain late in infection (20 dpi) overlap the size profile of smaller cells found in the lungs at the same time point. (C and D) Early after inoculation (3 dpi) the distributions of both *liv7*Δ mutant 1/2 and *ima1*Δ mutant 1/2 cells match the distribution of wild-type cells in the lungs (*n* = 3 mice, ≥50 cells per mouse). (E and F) At an early point in dissemination (14 dpi), *liv7*Δ mutant 1/2 cell populations were of larger average total cell diameter than wild-type C. neoformans cells in the lungs. *ima1*Δ mutant 1/2 cells were of smaller average total cell diameter than wild-type C. neoformans cells (*n* = 4 mice, ≥120 cells per mouse). *P* values were calculated using a Mann-Whitney test. Frequency bin size, 2.0 μm.

In wild-type-infected mice, cell and capsule sizes in the lungs had a broad distribution that shifted significantly over the course of infection, as observed by other investigators (53–55). Large cells were abundant early in infection, particularly at 3 dpi (Fig. 6A). These cells were likely Titan cells, which are large, highly polyploid, and increase their size and ploidy through nonmitotic genome replication (14). However, as infection progressed, the frequency of large cells decreased. By 20 dpi, smaller cells, around 10 μm in total diameter, dominated the lungs in number (Fig. 6A). The cell body size and capsule thickness distributions experienced proportional shifts such that overall the ratios of cell size to capsule thickness were maintained (Fig. S6A and B). In the brain, the distribution of cell and capsule sizes was much narrower and overlapped the population of smaller cells in the lungs (Fig. 6B).

When we compared the total cell diameter distributions of the wild type and the exo-GXM mutants in the lungs, there was no difference at 3 dpi (Fig. 6C and D). However, by an early time point in dissemination (14 dpi), the frequency of smaller cells was lower in *liv7*Δ mutant 1/2-infected mice and higher in *ima1*Δ mutant 1/2-infected mice than in wild-type-infected mice (Fig. 6E and F). These differences were not due to relative capsule size, as the ratios of cell size to capsule thickness were similar among all strains (Fig. S6C).

**FIG 7:**
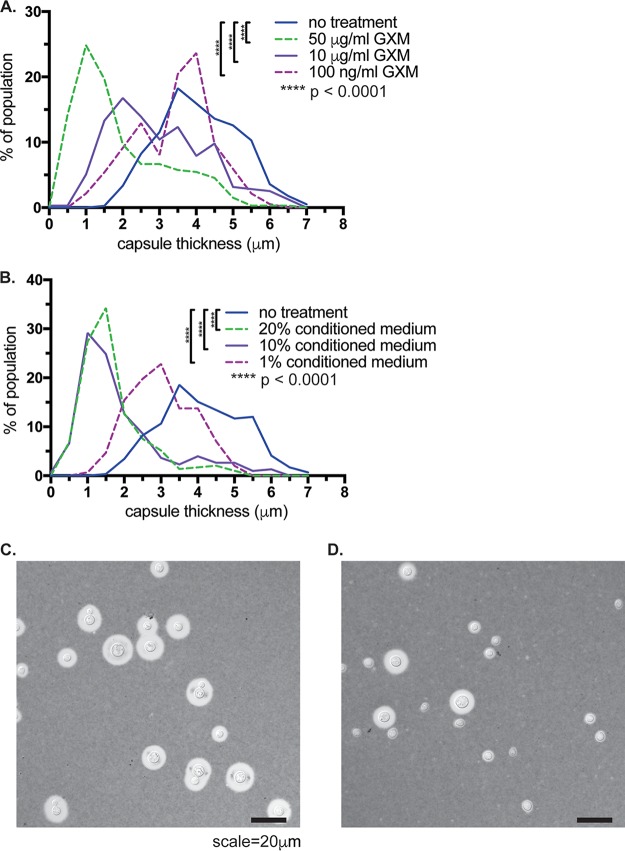
Treatment with GXM decreases capsule thickness. We induced cell surface capsule by growing cells for 24 h in 10% Sabouraud's medium, pH 7.3, and then added various concentrations of either purified GXM (A) or conditioned medium from a YNB-grown culture of wild-type (KN99) C. neoformans cells (B). We found a dose-dependent decrease in capsule thickness following exposure to both purified GXM and conditioned medium. Histograms contain data from four separate experiments, with at least 60 cells measured per condition for each experiment. We also observed a decrease in cell size (see Fig. S7 in the supplemental material) in cells treated with GXM or conditioned medium. *P* values were calculated using a Mann-Whitney test. Representative differential interference contrast images of untreated cells (C) or cells treated with 50 μg/ml GXM (D) are shown. Frequency bin size, 0.5 μm.

Due to this correlation between cell and capsule sizes and exo-GXM, we hypothesized that exo-GXM could influence cell and capsule sizes. To test this, we grew cells in strong cell surface capsule-inducing medium (10% Sabouraud's medium, pH 7.3) with minimal exo-GXM release. After 24 h of growth at 37°C, we diluted the cultures 1:2 in fresh medium and added 100 ng/ml, 10 μg/ml, or 50 μg/ml of purified GXM. After an additional 24 h of growth, we measured cell and capsule sizes. We found that both capsule thickness (Fig. 7A) and cell size (Fig. S7A) decreased in a dose-dependent manner. The greatest decrease was in capsule thickness, which showed a decrease from a median of 4 μm in control cultures to 1.5 μm in cultures treated with 50 μg/ml GXM, a decrease of 62.5%. A GXM dose of 100 ng/ml produced a more modest decrease, to a median capsule thickness of 3.6 μm (a 10% decrease). GXM treatments of 50 μg/ml and 10 μg/ml also resulted in a change in cell size, from a median of 6.0 μm for untreated cells to 4.5 μm and 5.3 μm, respectively. At 100 ng/ml, GXM did not produce a decrease in cell size (Fig. S7A), despite the observed change in capsule thickness (Fig. 7A).

GXM purification can result in contamination by detergents from the purification protocol (30). Thus, we performed the same experiment but added conditioned medium (from a YNB-grown culture) instead of purified GXM. A final concentration of conditioned medium of 20%, 10%, or 1% resulted in decreases in both capsule thickness and cell size (Fig. 7B and S7B). These capsule and cell size changes also depended on growth: if we did not add fresh medium along with purified GXM, capsule thickness and cell size did not change (Fig. S7C and D).

Altogether, these data suggest that changes to exo-GXM release observed *in vitro* can affect pathogenesis. Total exo-GXM released throughout infection correlated with decreased survival, increased fungal burden, and more rapid generation of smaller (haploid) cells in the lungs, which appear more likely to disseminate due to their dominant presence in extrapulmonary organs.

### Exo-GXM limits innate immune cell infiltration into the brain.

In human patients, cryptococcal meningoencephalitis is associated with a striking paucity of inflammation (9). The main driver of mortality, particularly in immunocompromised patients, is thought to be excessive fungal burden and GXM accumulation within the CNS, which leads to a devastating increase in intracranial pressure (10). C57BL/6NJ mice infected with the highly virulent KN99 strain display similarly limited brain inflammation, despite significant fungal presence. For instance, when we histologically compared the brains of KN99-infected mice to those of mock-infected animals, we could detect very little sign of infiltrating immune cells by hematoxylin and eosin (H&E) staining in KN99-infected mice, despite local presence of fungi (Fig. S8). This was true both early (14 dpi) (Fig. S8A and B) and late (21 dpi) (Fig. S8C and D) in disseminated infection. Considering the immunosuppressive nature of GXM, we hypothesized that it could very likely play a role in limiting brain inflammation during infection. We correspondingly reasoned that infection with *liv7*Δ mutant 1/2 cells might result in increased immune cell infiltration into the brain due to reduced exo-GXM secretion by *liv7*Δ mutant 1/2 cells.

**FIG 8:**
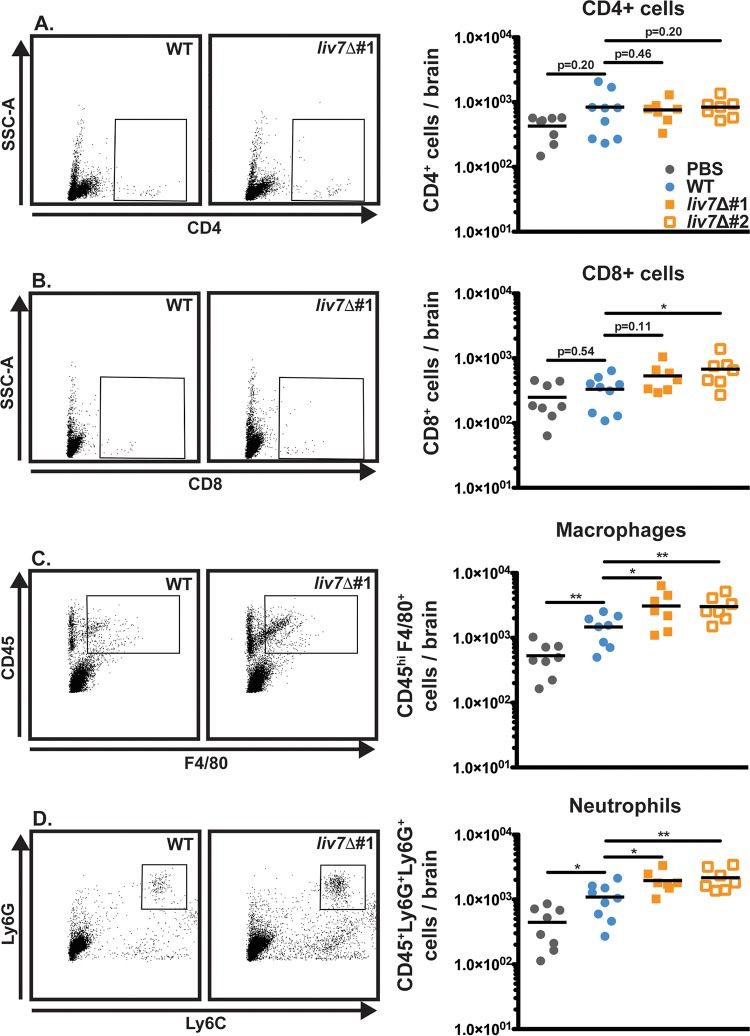
Mice infected with *liv7*Δ cells display increased innate immune cell infiltrate in the brain. Mouse brains were harvested late (20 dpi) in infection for flow cytometry analysis of infiltrating immune cells. (A) CD4^+^ T cells are scarce in both wild-type- and *liv7*Δ mutant 1/2-infected brains. (B) CD8^+^ T cells show a significant increase over wild-type in *liv7*Δ mutant 2-infected brains, but this was not replicated in *liv7*Δ mutant 1-infected brains. Macrophages (CD45^hi^ F4/80^+^) (C) and neutrophils (CD45^+^ Ly6G^+^ Ly6C^+^) (D) are significantly increased in the brains of *liv7*Δ mutant 1/2-infected mice compared to levels in wild-type- and mock-infected brains. *P* values were calculated using a Mann-Whitney test; bars represent the medians.

In order to address this hypothesis, we harvested the brains of wild-type-infected and *liv7*Δ mutant 1/2-infected animals at 20 days post-intranasal inoculation and analyzed immune cell infiltration into the brain via flow cytometry. CD4^+^ (Fig. 8A) and CD8^+^ (Fig. 8B) cells were scarce in both wild-type- and *liv7*Δ mutant 1/2-infected brains. These data suggest that T cells do not significantly respond to brain invasion by C. neoformans. Innate immune cells (macrophages/neutrophils) did show some response to wild-type C. neoformans cells in the brain, but the level was only slightly elevated compared to that in mock-infected animals (Fig. 8C and D). This is in stark contrast to bacterial or viral meningitis, which often shows high levels of infiltrating neutrophils and macrophages (7, 8). Infiltration of both macrophages and neutrophils was increased in *liv7*Δ mutant 1/2-infected brains (Fig. 8C and D). These results suggest that exo-GXM likely plays an important role in brain immunosuppression that is independent of surface capsule.

**FIG 9:**
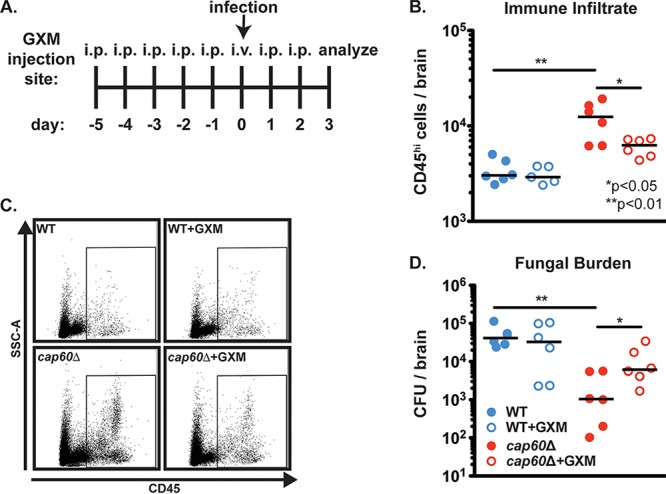
Purified GXM is sufficient to suppress immune cell infiltration into the brain in response to inflammation-inducing acapsular (*cap60*Δ) C. neoformans. Six-week-old C57BL/6NJ mice were intracranially inoculated with 200 *cap60*Δ fungal cells in 30 μl of 1× PBS. Beginning 5 days prior to inoculation, mice were administered intraperitoneal injections of either 200 μg/ml GXM or 200 μl of sterile PBS. On the day of inoculation, mice were administered this treatment intravenously to ensure that GXM would be present in the bloodstream. At 3 dpi brains were harvested to determine fungal burden by CFU counts. Separate mice were sacrificed to analyze infiltrating immune cells by flow cytometry. (A) Diagram of experimental procedures. i.p., intraperitoneal. (B) Mice infected with the *cap60*Δ mutant displayed increased brain immune cell infiltrate (CD45^hi^ cells) over wild-type-infected mice. Immune cell infiltration into the brains of *cap60*Δ mutant-infected mice was reduced with the administration of GXM. (C) Representative flow plots for data shown in panel B. (D) Mice infected with wild-type KN99 cells suffered increased fungal brain burden compared to the level in mice infected with the *cap60*Δ mutant. Administration of GXM had no significant effect on wild-type fungal burden but resulted in significantly increased *cap60*Δ mutant fungal burden compared to that of *cap60*Δ mutant-infected mice that did not receive GXM. *P* values were calculated using a Mann-Whitney test; bars represent the medians.

We next sought to determine if exo-GXM was sufficient to suppress immune cell infiltration into the brain if we induced brain inflammation by direct intracranial inoculation. We purified GXM from YNB-grown cultures using standard methods (30). Since we detected GXM associated with the brain up to 5 days prior to the appearance of CFU (Fig. S5), we administered 200 μg of purified GXM daily by intraperitoneal injection beginning 5 days prior to inoculation (Fig. 9A). Additional mice were administered sterile PBS as a control. We then inoculated mice intracranially with either wild-type KN99 or acapsular *cap60*Δ cells. Unsurprisingly, *cap60*Δ cells elicited greater numbers of immune cells infiltrating the brain (Fig. 9B and C) and achieved a significantly lower fungal burden (Fig. 9D) than wild-type *C. neoformans*. However, administration of GXM to mice infected with *cap60*Δ cells reduced immune cell infiltration (cells expressing CD45 at high levels [CD45^hi^]) into the brain (Fig. 9B and C and S9) and increased fungal burden compared to that of PBS-treated mice (Fig. 9D). These results demonstrate that in the context of an inflammatory infection, exo-GXM is sufficient to promote fungal survival in the brain, likely through the suppression of brain immune cell infiltration.

## DISCUSSION

Surface capsule is critical for C. neoformans virulence. However, GXM that is not attached to the cell surface, or exo-GXM, accumulates to significant levels in laboratory culture and during infection (10, 30, 31). Our data strongly suggest that C. neoformans inversely regulates surface capsule formation and exo-GXM release according to environmental cues. Under our tested conditions, GXM was constitutively produced but was alternately retained at the cell surface or released into the extracellular milieu (Fig. 1). We also observed that *O*-acetylation of GXM's mannose backbone changes with environmental conditions (see Fig. S1 in the supplemental material). Previous findings have also indicated that exo-GXM release might be an active process. For instance, a study comparing the properties of exo-GXM and capsular GXM showed that, despite their identical sugar compositions, capsular GXM and exo-GXM manifested distinct biophysical and antigenic properties (34).

Differential regulation of surface capsule and exo-GXM could occur at the level of GXM polymer length and/or other modifications (35–37). Another possibility is that exo-GXM release is regulated by changing levels of alpha-glucan, which anchors GXM to the cell surface (56), or by altering release of exosomes containing GXM (57). Either way, changes in GXM levels, distribution, and modification are likely to influence disease progression (19, 40, 41). More work is required to elucidate biophysical differences between cell surface-retained GXM and exo-GXM.

We identified genes that play a role in exo-GXM release. Deletion of *LIV7*, which decreases virulence in mice, reduces exo-GXM release in mild or non-capsule-inducing growth medium but does not affect capsule thickness. It has been previously demonstrated that *LIV7* is important for virulence and likely functions in Golgi transport (42, 44). In contrast, deletion of the gene *IMA1* increased virulence in mice, potentially by increasing exo-GXM release under strong capsule-inducing conditions without affecting capsule thickness. We used these two exo-GXM mutants as tools to investigate the biological importance of exo-GXM independent of surface capsule.

The *liv7*Δ mutant was identified as having a competitive disadvantage in a pool of gene deletion mutants (42), which would be surprising for a cell-extrinsic factor such as exo-GXM, whose effects might be muted in a competitive assay. The *ima1*Δ mutant was not found to have altered virulence in this screen. One interpretation is that *LIV7* has an unidentified phenotype in addition to exo-GXM release that impacts virulence. However, *liv7*Δ cells did not exhibit any significant (∣Z∣ ≥ 2.5) phenotype when grown in the presence of over 150 different small molecules in a chemical-genetics screen (58). Other interpretations could include that individual fungal cells end up in separate sections of the lungs or that they are phagocytosed by different macrophages, creating localized infection conditions where one mutant might not mask the phenotype of another. Each pool screened by Liu et al. contained, on average, more than one capsule-deficient mutant (42), which would also lower the total exo-GXM levels.

Ours is not the first study connecting various levels of exo-GXM release with virulence. Analysis of a virulence-associated transcriptional network map previously revealed a positive correlation with *in vitro* exo-GXM release and mouse lung infectivity over 7 days (59). However, the transcription factor mutants also had altered surface capsule thickness, which may have influenced infectivity (59). Deletion of the flippase-encoding gene *APT1* also resulted in reduced *in vitro* exo-GXM release despite wild-type surface capsule. The knockout was hypovirulent but, in contrast to the *liv7*Δ and *ima1*Δ mutants, had reduced surface capsule thickness *in vivo* (60). Our results support these previous findings and provide a clearer link between exo-GXM and virulence because these mutants do not suffer alterations to any additional virulence factors that we screened. Our data also provide additional support for a model in which regulated release of exo-GXM enhances virulence independent of surface capsule.

In a murine infection model, we showed a correlation among elevated *in vitro* exo-GXM levels, fungal burden, and poor host survival. Exo-GXM levels also vary considerably among humans suffering from cryptococcal meningoencephalitis, with increased exo-GXM levels predictive of a nonprotective Th2 immune signature and increased mortality (32, 61). These correlations suggest that perturbing exo-GXM release by cryptococcal cells appreciably affects the progression of infection since differences between wild-type- and *liv7*Δ-infected mice are apparent in mid to late, not early, infection (Fig. 5C). Work on both Aspergillus fumigatus and Candida albicans has demonstrated that mutant strains capable of disease initiation are not necessarily capable of establishing robust disease (62). The opposite is also true: genes necessary for later disease might not have a defect in the initial establishment of infection (63). We propose that exo-GXM is more likely involved in establishment and dissemination rather than disease initiation. We also established a positive correlation between exo-GXM release and biofilm adherence, suggesting that exo-GXM release during environmental growth may be important for promoting community-level structure and adherence. It would not be surprising for there to be additional functions for exo-GXM in environmental settings.

Interestingly, exo-GXM levels also correlated with changes in cell body and capsule size distributions in the lungs. In C. neoformans-infected animals, fungal cell body size and capsule thickness decreased over the course of infection as exo-GXM levels increased. Correspondingly, increased GXM levels in the mouse lungs positively correlated with an increased frequency of smaller cells at an early time point in dissemination. C. neoformans cells in the brain and other extrapulmonary organs are much smaller than those in the lungs (Fig. 6B) (46, 50, 54), suggesting that the emergence of smaller cells in the lungs is an important step in dissemination. The addition of purified GXM to C. neoformans cells growing in capsule-inducing medium was sufficient to decrease cell body size and capsule thickness in a growth-dependent manner (Fig. 7). These data suggest that exo-GXM may actually provide a concentration-dependent signal to C. neoformans cells that reduces cell size and capsule thickness. In the lungs, this mechanism may be a contributing factor in the generation of small cells with a greater propensity for dissemination.

These data raise the question of how C. neoformans cells might sense a polymer that also covers its cell surface. Since GXM is modified by *O*-acetylation (41) and can contain different branching structures (36), one possibility is that exo-GXM and cell surface GXM are differentially modified and that the fungal cell senses these modifications and then responds by mechanisms similar to two-component signal transduction systems in bacteria. This will be an area of future study. Overall, our results suggest that exo-GXM is an actively secreted virulence factor that may influence cell morphology to facilitate dissemination and is capable of distally suppressing immune cell infiltration into the brain.

There is a large body of literature demonstrating immunosuppressive properties for GXM (19, 64). We focused on the brain as cryptococcal meningoencephalitis is the leading cause of death in cryptococcosis patients and is characterized by low levels of inflammation (9). Here, we observed that deleting a gene required for wild-type levels of exo-GXM release *in vitro* (*LIV7*) altered the host immune response to C. neoformans brain infection. Mice infected with *liv7*Δ cells had increased numbers of macrophages and neutrophils infiltrating the brain compared to levels in wild-type-infected mice. Furthermore, administration of purified GXM was sufficient to reduce the number of brain-infiltrating immune cells in the context of acapsular C. neoformans infection. Our data suggest that removing exo-GXM from infected patients by antibody treatment could aid cryptococcosis treatment.

## MATERIALS AND METHODS

### Conditioned medium collection.

C. neoformans cells were cultured overnight in YNB with 2% glucose at 30°C before being subcultured at 1:100 in the desired medium. Cultures were read at an optical density at 600 nm (OD_600_) 24 h later, and values were normalized to the lowest measured OD_600_ value. Cells were pelleted by centrifugation at 3,000 × *g* for 5 min. The supernatant was collected and passed through a 0.22-μm-pore-size filter, yielding conditioned medium.

The following growth media were used in this study: YPAD (20 g/liter Bacto peptone, 10 g/liter Bacto yeast extract, 2% glucose, 0.4 g/liter adenine sulfate); YPD (20 g/liter Bacto peptone, 10 g/liter Bacto yeast extract, 2% glucose); YNB (yeast nitrogen base without amino acids [catalog no. 291940; Difco], 2% glucose); 25% YNB plus 2% glucose; low-iron medium (LIM) (5 g/liter asparagine, 0.4 g/liter K_2_HPO_4_, 0.1 g MgSO_4_·7H_2_O, 0.5 mg/liter thiamine, 0.029 mg/liter boric acid, 1.88 mg/liter CuSO_4_·5H_2_O, 0.36 mg/liter MnCl_2_·4H_2_O, 0.021 mg/liter ZnCl_2_, 0.18 mg/liter NaMoO_4_·2H_2_O, 0.05 mg/liter CaCl_2_·2H_2_O, 0.05 mM bathophenanthroline disulfonic acid [BPDS], 1 mM EDTA, 2% glucose, 50 mM morpholinepropanesulfonic acid [MOPS], pH 6.0); 10% Sabouraud's dextrose medium (catalog no. 238230; Difco) buffered with 50 mM HEPES (pH 8.0), HEPES (pH 7.3), MOPS (pH 6.0), or morpholineethanesulfonic acid (MES; pH 5.0); yeast carbon base ([YCB] catalog no. 239110; Difco) plus 5 g/liter urea; YCB plus 0.5 g/liter urea.

### Conditioned medium blots.

Ten microliters of conditioned medium collected from C. neoformans cultures was loaded onto a 0.6% agarose gel and run at 33 V for 18 to 20 h with 0.5× Tris-borate-EDTA (TBE). The gels were processed with a 10-min depurination rinse in a 0.25 M HCl solution, followed by a 30-min denaturation incubation in a solution of 1.5 M NaCl–0.5 M NaOH and a 30-min neutralization incubation in 1.5 M NaCl–0.5 M Tris-HCl, pH 7.5. The gels were rinsed in distilled water following each incubation. Gel contents were subsequently transferred to a positively charged membrane using a standard Southern blot protocol with 10× SSC (1× SSC is 0.15 M NaCl plus 0.015 M sodium citrate) in the reservoir. After overnight transfer, the blots were soaked briefly in 2× SSC and dried. Blots were then blocked for 1 h in 1× PBS–5% milk and incubated with shaking overnight at 4°C in 1× PBS–5% milk with anti-GXM monoclonal antibody at 1:40,000. The following morning, blots were rinsed three times in 1× PBS, incubated for 2 h in 1× PBS–5% milk with 1:2,500 goat anti-mouse horseradish peroxidase (HRP) antibody, and washed for 2.5 h in 1× PBS–0.1% Tween 20, with the wash buffer changed every 20 min. For imaging, blots were developed with Clarity Western ECL substrate (catalog no. 170-5061; Bio-Rad) and imaged on a Bio-Rad Western blot imager. Anti-GXM monoclonal antibodies used in this study were F12D2 and 1326 (Thomas Kozel, University of Nevada, Reno, NV).

### Cell measurements.

C. neoformans cells collected from laboratory media were spun down at 3,000 × *g* for 5 min, washed twice in 1× PBS, and resuspended in 1× PBS. To collect cells from infected mouse organs, 1 ml of organ homogenate was passed through a 70-μm-pore-size cell strainer (catalog no. 22-363-548; Fisher). At this junction, capsule measurement methods were the same for both laboratory-grown and mouse-isolated C. neoformans cells. Cells were fixed for 15 min in 2% paraformaldehyde, washed twice with 1× PBS, and then resuspended in 100 μl of 1× PBS. Four microliters of cell suspension was mixed with 4 μl of india ink (catalog no. 44201; Higgins) on a microscope slide, coverslipped, and visualized. Successive, representative pictures were taken from the outside of the coverslipped area toward the middle because smaller cells tended to spread toward the edges of the coverslip more so than larger cells. Total cell diameter was measured as the distance from one edge of the capsule to the opposite edge.

Cell body diameter was measured as the distance from one edge of the cell wall to the opposite edge. Capsule thickness was calculated as follows: (total cell diameter − cell body diameter)/2.

### Screen for exo-GXM mutants.

Cells were spotted from 96-well frozen stocks to OmniTrays containing YPD agar and then grown for 48 h at 30°C. Colonies were used to inoculate deep-well plates containing 1 ml yeast nitrogen base (YNB) per well. Deep-well plates were grown at 37°C for 48 h with shaking (280 rpm). Ten microliters of YNB culture was then used to inoculate cultures grown on 10% Sabouraud's medium (pH 7.3), which were then grown at 37°C for 48 h with shaking. After growth, all cultures, grown on either YNB or 10% Sabouraud's medium, pH 7.3, were harvested by centrifugation, and then the supernatant was collected and stored for analysis.

We analyzed exo-GXM in YNB supernatants by dot blotting 4 μl of supernatant into each well of a dot blotter containing positively charged nylon membrane presoaked in 2×SSC and then applying a vacuum. Membranes were air dried and then blocked and incubated with anti-GXM F12D2 antibody using standard procedures (see “Conditioned medium blots” above). 10% Sabouraud's medium conditioned medium samples were run on agarose gels and transferred to nylon membranes (see “Conditioned medium blots” above).

Once we identified mutants with altered exo-GXM levels decreased in YNB cultures or increased in 10% Sabouraud's medium (pH 7.3) cultures, we grew all mutants in 10% Sabouraud's medium (pH 7.3) and then measured capsule thickness. Mutants with decreased cell surface capsule thickness (approximately 25% decrease compared to the thickness of wild-type cells) were eliminated from further analysis. We then repeated the growth and exo-GXM blot assays for each strain. We normalized for cell density (to account for slow-growing mutants), filtered the conditioned medium through a 0.22-μm-pore-size filter to remove cells, and ran 10 μl of conditioned medium on an agarose gel using the procedure described above (see “Conditioned medium blots”). Finally, we stained for exposure of PAMPs such as chitin and mannoprotein (see “Lectin staining” below) and removed mutants with increased exposure.

### Lectin staining.

Cells grown for 24 h in the appropriate medium were pelleted, washed twice in 1× PBS, and fixed for 12 min in 2% paraformaldehyde. Cells were then washed twice in 1× PBS and resuspended in 1× PBS. To an aliquot of cells, wheat germ agglutinin (WGA) conjugated to fluorescein (catalog no. FL-1021; Vector Laboratories) was added to a final concentration of 5 μg/ml, and samples were incubated for 30 min at room temperature with shaking. At the end of the WGA incubation, concanavalin A (ConA) conjugated to rhodamine (catalog no. RL-1002; Vector Laboratories) was added to a final concentration of 50 μg/ml. Cells were washed once in 1× PBS and imaged immediately.

### Melanization and urease secretion.

Cells grown overnight in YNB medium were washed twice in 1× PBS and resuspended to a final concentration of 2.5 × 10^6^ cells/ml in 1× PBS. Ten microliters of cell suspension was spotted onto l-3,4-dihydroxyphenylalanine (l-DOPA)-containing agar or Christensen's urea agar (catalog no. 27048; Sigma). Plates were checked daily for changes in melanization (brown/black colonies on l-DOPA) and urease secretion (pink area surrounding colonies on Christensen's urea).

### GXM purification.

GXM was purified as described previously (30). Briefly, 100 ml of C. neoformans cells was cultured in YNB medium plus 2% glucose for 5 days at 30°C. Cultures were centrifuged at 12,000 × *g* for 15 min, and the supernatant was collected. Polysaccharides were precipitated from the supernatant overnight with the addition of 3 volumes of 95% ethanol (EtOH) at 4°C. The solution was then centrifuged at 15,000 × *g* for 1 h, resuspended in 0.2 M NaCl, and sonicated. After sonication, 3 mg of hexadecyltrimethylammonium bromide (CTAB) (catalog no. 227160; Fisher) per 1 mg of precipitate was slowly added to the solution on low heat. After samples were removed from the heat, another 2.5 volumes of 0.05% CTAB was added. The solution was centrifuged at 11,000 × *g* for 2 h, and the pellet was washed in 10% EtOH to remove any remaining CTAB. After an additional centrifugation at 18,000 × *g*, the pellet was resuspended in 1 M NaCl and sonicated for 2 h. Once the GXM was solubilized, it was dialyzed (3.5-kDa cutoff) versus sterile distilled water and then lyophilized. Purified, lyophilized GXM was stored at −80°C for subsequent use.

### Adherence assay.

We used a slightly modified protocol of biofilm formation and XTT [2,3-bis-(2-methoxy-4-nitro-5-sulfophenyl)-2*H*-tetrazolium-5-carboxanilide] analysis, as described previously (48, 49). Briefly, 5-ml cultures were grown overnight in YNB with 2% glucose at 30°C, pelleted, washed in 1× PBS, and resuspended in 1× PBS. Cells were counted on a hemocytometer, diluted to 10^7^ cells/ml in the appropriate medium, and plated in 100-μl volumes in 96-μl polystyrene plates (avoiding edge wells). Sterile medium was plated as a negative control. Plates were incubated for 48 h at 37°C to allow for adherence and biofilm maturation. Plates were then washed three times with 200 μl of 1× PBS–0.05% Tween 20 using a BioTek 405 TS microplate washer set to an intermediate flow rate. To determine the relative levels of cells that remained after washing, we used an XTT reduction assay to quantitate metabolic activity as a proxy for viable cell density. After plate washing, 100 μl of a solution containing 0.5g/liter XTT (catalog no. X6493; Fisher) and 4 μM menadione (catalog no. 58-27-5; Sigma) in acetone in 1× PBS was added to each well. Menadione was added to fresh XTT solution immediately prior to addition of the solution to a plate. Plates were incubated for 5 h before aliquots of 80 μl of supernatant were moved to a new plate for absorbance reading at 490 nm.

### Mice.

For the intranasal infection model, we used ∼8-week-old female C57BL/6NJ mice (Jackson Laboratory). C. neoformans cells were harvested from cultures grown overnight in YPD medium at 30°C, washed two times in 1× PBS, resuspended in 1× PBS, and then counted with a hemocytometer to determine the inoculum. Mice were anesthetized with ketamine/dexmedetomidine hydrochloride (Dexdomitor) (milligrams/gram) intraperitoneally before they were spread on horizontally tied thread by their front incisors. Mice were kept warm with a heat lamp and inoculated intranasally with 2.5 × 10^4^ C. neoformans cells in 50 μl of 1× PBS. After 10 min, mice were removed from the thread and administered the reversal agent atipamezole (Antisedan; ∼0.0125 mg/g) intraperitoneally. For survival analyses, mice were weighed daily and euthanized by CO_2_ asphyxiation and cervical dislocation when they lost 15% of their initial mass. Mice used to analyze fungal burden, capsule size, and GXM levels were euthanized by the same measures at designated time points. Mice used for flow cytometry analysis were anesthetized with isoflurane and intracardially perfused with cold 1× PBS before cervical dislocation and brain extraction.

For the intracranial infection model, we used ∼6-week-old female C57BL/6NJ mice (Jackson Laboratories). C. neoformans inoculum was prepared as described above. Prior to inoculation, mice were anesthetized with ketamine/dexmedetomidine hydrochloride as described above. Mice were inoculated intracranially with 200 C. neoformans cells in 30 μl of 1× PBS via a 26-gauge 1/2-in. needle. Animals were then administered atipamezole to speed recovery. All animal procedures were approved by the University of Utah Institutional Animal Care and Use Committee.

### Fungal burden.

Organs were harvested from euthanized mice, placed on ice, and homogenized with a Tissue Master Homogenizer (Omni International) in 5 ml of 1× PBS. Serial dilutions of organ homogenates were plated on Sabouraud's dextrose agar with 10 mg/ml gentamicin and 100 mg/ml carbenicillin and stored at 30°C in the dark for 3 days. Resulting CFU were then counted to determine fungal burden.

### GXM ELISA.

Five hundred microliters of the same mouse organ homogenate used for CFU counts and C. neoformans cell measurements was collected and spun down at 3,000 × *g* for 5 min. The supernatant was then passed through a 0.22-μm-pore-size filter to remove cells. GXM levels in the resulting filtrate were quantified using an Alpha Cryptococcal Antigen enzyme immunoassay (CRY101; IMMY). GXM purified from C. neoformans cultures was diluted to generate standard curves.

### Histology.

Perfused mouse brains were divided in half and fixed overnight in 4% paraformaldehyde. Sagittal slices (8 μm thick) were mounted on microscope slides and stored at −20°C. Successive sections were stained with hematoxylin and eosin or Grocott's methenamine silver (catalog no. 87008; ThermoFisher Scientific).

### Flow cytometry.

Perfused mouse brains were collected in RPMI medium, ground gently to disperse tissue, and spun in 90% Percoll (P1644; Sigma) with a 63% Percoll underlay to isolate leukocytes at the interface. Leukocytes were resuspended in fluorescence-activated cell sorting (FACS) buffer (1× PBS, 1% bovine serum albumin) and stained with the appropriate fluorescently labeled antibodies. Labeled cells were fixed for 20 min in 4% paraformaldehyde before analysis on an LSRFortessa instrument (BD Biosciences). Antibodies used in this study (eBiosciences) were the following: CD45-efluor450 (catalog no. 48-0451-82), CD4-allophycocyanin (APC) (17-0041-82), CD8-fluorescein isothiocyanate (FITC) (11-0081-82), F4/80-FITC (11-4801-82), Ly6G-FITC (11-5931-82), and Ly6C-APC (17-5932-82).

## Supplementary Material

Supplemental material
