# Supplementary material for: Regulated Release of Cryptococcal Polysaccharide Drives Virulence and Suppresses Immune Cell Infiltration into the Central Nervous System
Source: Infect Immun. 2018 Feb 20;86(3):e00662-17. doi: 10.1128/IAI.00662-17 (PMC5820953; doi:10.1128/IAI.00662-17)
Supplement: Supplemental material [file supp_86_3_e00662-17__index.html]

Supplemental material 

# Regulated Release of Cryptococcal Polysaccharide Drives Virulence and Suppresses Immune Cell Infiltration into the Central Nervous System

## Supplemental material

- Supplemental file 1 -

  Legend for Table S1. Fig. S1. Proportion of *O*-acetylated exo-GXM increases under stronger capsule-inducing conditions. Fig. S2. Canonical virulence determinants are intact in *liv7*Δ and *ima1*Δ cells. Fig. S3. Liver and spleen fungal burden mostly correlates with *in vitro* exo-GXM production. Fig. S4. Total free GXM levels in mice infected with *C. neoformans* exo-GXM mutants. Fig. S5. GXM appears in brains and spleens prior to the appearance of CFU. Fig. S6. Distribution of *C. neoformans* cell body diameter and cell capsule thickness shift over the course of lung infection. Fig. S7. Treatment with GXM decreases cell size. Fig. S8. Few immune cells infiltrate the brains of mice with disseminated cryptococcosis, despite high fungal burden. Fig. S9. Administration of purified GXM to mice inoculated intracranially with acapsular *C. neoformans* reduces brain immune infiltration.

  PDF, 2.0M
- Supplemental file 2 -

  Table S1. Exo-GXM mutant screen results.

  XLSX, 34K
