# Supplementary material for: Regulated Release of Cryptococcal Polysaccharide Drives Virulence and Suppresses Immune Cell Infiltration into the Central Nervous System
Source: Infect Immun. 2018 Feb 20;86(3):e00662-17. doi: 10.1128/IAI.00662-17 (PMC5820953; doi:10.1128/IAI.00662-17)
Supplement: Supplemental material [file IAI.00662-17_zii999092299s1.pdf]

Table S1: Exo-GXM mutant screen results. We screened the *C. neoformans* partial knockout collection (CM18 background, 1200 targeted gene knockouts) under YNB, which results in high exo-GXM release by wild-type cells, or 10% Sabouraud's pH7.3, which results in low exo-GXM release. **(A)** Gene deletions which resulted in reduced exo-GXM release in YNB after 24 hours but no growth defect or a substantial reduction (>25%) reduction in cell surface capsule thickness in 10% Sabouraud's pH 7.3. **(B)** Gene deletions which resulted in increased exo-GXM release in 10% Sabouraud's pH 7.3 after 24 hours. Class 1 gene deletion mutants had approximately wild-type-sized capsule thickness, while Class 2 mutants had reduced capsule thickness in 10% Sabouraud's. pH 7.3.

Figure S1: Proportion of *O*-acetylated exo-GXM increases under stronger capsule inducing conditions. Conditioned media was collected and blotted as in **Fig. 1**. **(A)** Detection of GXM with an acetylation insensitive mAb (F12D2). **(B)** Detection of GXM from the same conditioned media with an acetylation-sensitive mAb (1326), which only recognizes *O*-acetylated GXM. Increased intensity indicates a greater level of *O*-acetylated GXM.

Figure S2: Canonical virulence determinants are intact in *liv7*Δ and *ima1*Δ cells. **(A)** Cells were grown overnight in YNB+2%glucose, stained with the fluorescently labeled lectins concanavalin A (ConA-rhodamine) and wheat germ agglutinin (WGA-fluorescein) to estimate exposure of PAMPs on the cell surface. Concanavalin A recognizes mannoproteins and wheat germ agglutinin recognizes chitin. **(B)** Cells were

grown overnight in YNB+2%glucose, subcultured 1:100 in 10% Sabouraud's dextrose (10% Sab), pH 7.3, and stained as in **(A)**. PAMP exposure was similar across all strains except *cap60*Δ, which lacks surface capsule. **(C)** $2.5x10^4$ cells were spotted on L-3,4-dihydroxyphenylalanine (L-DOPA) agar to observe melanization 48 hours later. No obvious differences were detected. **(D)** $2.5x10^4$ cells were spotted on Christensen's urea agar to observe urease secretion 48 hours later as the change in agar coloration from orange to pink. No obvious differences were detected.

Figure S3: Liver and spleen fungal burden mostly correlates with *in vitro* exo-GXM production. (A) 8-week-old female C57BL/6NJ mice were inoculated intranasally with $2.5x10^3$ cryptococcal cells. *ima1*Δ#1 / #2 (n=10 and n=10, respectively) reach endpoint significantly sooner than wild-type infected mice (n=10, p). Wild-type infected mice reached endpoint sooner than *liv7*Δ#1 / #2 (n=10 and n=10, respectively) infected mice. P-values were calculated using a Log-rank (Mantel-Cox) Test. **(B)** 8-week-old female BALB/cJ mice were inoculated intranasally with $2.5x10^4$ cryptococcal cells. *ima1*Δ#1 / #2 (n=10 and n=10, respectively) reach endpoint significantly sooner than wild-type infected mice (n=10). Wild-type infected mice reached endpoint sooner than *liv7*Δ#1 / #2 (n=10 and n=10, respectively) infected mice. P-values were calculated using a Log-rank (Mantel-Cox) Test. Panels C-F are from the same experiments as in **Fig. 5**. **(C)** Fungal burden in the livers of wild-type and *liv7*Δ#1 / #2 infected mice did not show consistent differences over the course of infection. **(D)** Fungal burden in the spleens of *liv7*Δ#1 / #2 infected mice was significantly lower than wild-type-infected mice at 10 and 17 dpi. **(E)** Fungal burdens in *ima1*Δ#1 / #2 infected livers were

significantly higher than wild-type at 14 and 16 dpi. **(F)** Fungal burdens in *ima1*Δ#1 / #2-infected spleens were significantly higher than wild-type infected mice at 14 and 16 dpi. P-values were calculated using a Mann-Whitney test.

Figure S4: Total free GXM levels in mice infected with *C. neoformans* exo-GXM mutants. Tissues from mice in **Fig. 3B-G** were homogenized and passed through a 0.22 μm filter to remove fungal cells, then GXM levels were measured by ELISA. **(A)** Mouse lungs infected with *liv7*Δ#1 / #2 cells showed trends toward reductions in exo-GXM levels when compared to wild-type, though statistical significance is not consistent across independent gene deletions. **(B and C)** Mouse livers and spleens infected with *liv7*Δ#1 / #2 cells showed reduced exo-GXM levels when compared to wild-type-infected organs at 14 dpi. **(D)** Mouse brains infected *liv7*Δ#1 / #2 cells showed reduced exo-GXM when compared to wild-type at 17dpi. **(E-G)** Exo-GXM was increased in *ima1*Δ#1 / #2 infected lungs, livers, and spleens when compared to wild-type-infected organs at 14dpi. **(H)** No significant differences in exo-GXM were observed in *ima1*Δ#1 / #2 infected brains when compared to wild-type-infected brains. P-values were calculated using a Mann Whitney t test.

Figure S5: GXM appears in brains and spleens prior to the appearance of CFU. A time course of **(A)** GXM per CFU (lungs, liver, spleen, and brain), **(B)** GXM per CFU (lungs), **(C)** fungal burden (CFU), and **(D)** GXM per organ following infection with wild-type *C. neoformans.* GXM is detectable in all organs by 3 dpi. CFU were not detectable

in brains or spleens until 10 dpi. These data are the compiled wild-type infection data from **Fig. 5**, **Fig. S3**, and **Fig. S4**.

Figure S6: Distribution of *C. neoformans* cell body diameter and cell capsule thickness shift over the course of lung infection: These data are from the same experiments as **Fig. 6**. **(A)** Average *C. neoformans* cell body diameter in the lungs decreases over the course of infection (n=3-4 mice per time point, ≥120 cells per mouse). **(B)** Average capsule thickness in the lungs decreases over the course of infection at a rate similar to the change in cell body diameter (n=3-4 mice per time point, ≥120 cells per mouse). **(C)** The proportion of cell size to capsule thickness in the lungs is similar across wild-type, *liv7*∆#1 / #2, and *ima1*∆#1 / #2 cells in the lungs (n=4 mice, ≥120 cells per mouse). (Frequency bin size = 2.0) P-values were calculated using a Mann-Whitney test; error bars show medians.

Figure S7: **Treatment with GXM decreases cell size.** These data are from the same experiments as **Fig. 7**. Cell size decreases in a dosage-dependent manner with the addition of (**A**) purified GXM at 50 µg/ml or 10 µg/ml, but not 100 ng/ml, even though capsule thickness decreased with the addition of 100 ng/ml GXM. (**B**) Conditioned medium at final concentrations of 20%, 10%, or 1% all decrease cell size. Capsule thickness and cell size do not change if cultures are not administered additional growth medium (10% Sabouraud's, pH 7.3) along with (**C**) purified GXM or (**D**) conditioned medium, suggesting that these size changes are growth-dependent. P-values were calculated using a Mann-Whitney test.

Figure S8: Few immune cells infiltrate the brains of mice with disseminated cryptococcosis, despite high fungal burden. **(A)** Representative hematoxylin and eosin (H&E) and **(B)** consecutive Grocott's methenamine silver (GMS) stained midbrain sections early (14 dpi) in brain infection. We observed no signs of inflammatory infiltrate (excess purple hematoxylin staining) and minimal fungal presence (black silver staining; arrows point to fungi) early. **(C)** Representative H&E and **(D)** GMS stained cerebral cortex sections late (21 dpi) in brain infection. We continued to detect few signs of inflammatory infiltrate in H&E stained sections late in infection, despite significant and diffuse fungal presence within the meninges and parenchyma of the brain (arrows point to fungi).

Figure S9: Administration of purified GXM to mice inoculated intracranially with acapsular *C. neoformans* reduces brain immune infiltration. These data are from the same experiments as **Fig. 10**. Brain infiltrating immune cells were detected by flow cytometry and broken into **(A)** $CD45^{hi}F4/80^{+}$ macrophages, **(B)** $CD45^{+}Ly6G^{+}Ly6C^{+}$ Neutrophils, **(C)** $CD4^{+}$ (T cells), **(D)** $CD8^{+}$ (T cells). P-values were calculated using a Mann-Whitney test.

Figure S1

A. F12D2
O-acetyl (+) GXM binding
O-acetyl (-) GXM binding

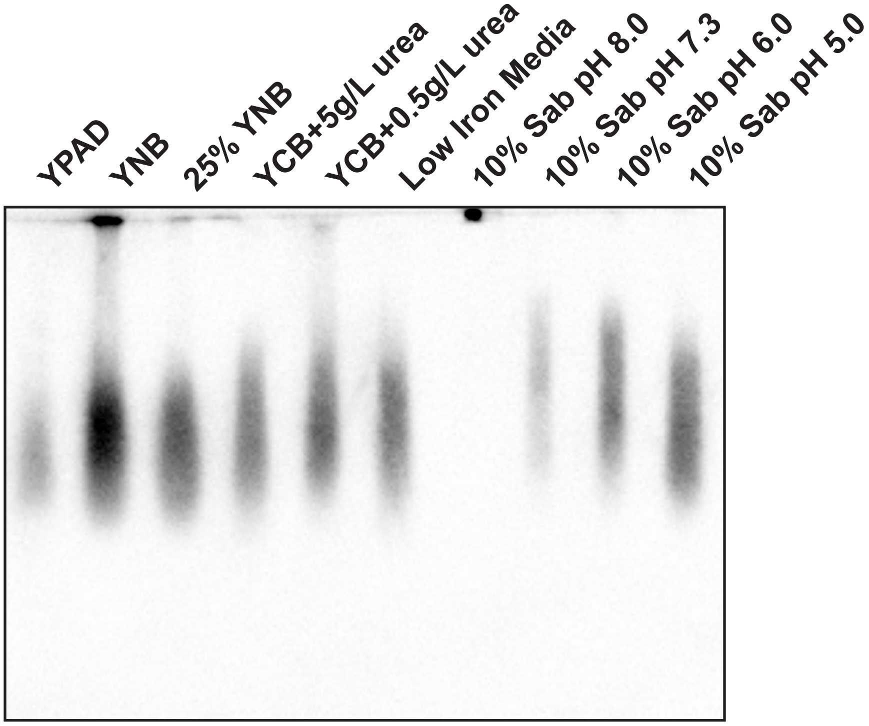

B. 1326
O-acetyl (+) GXM binding

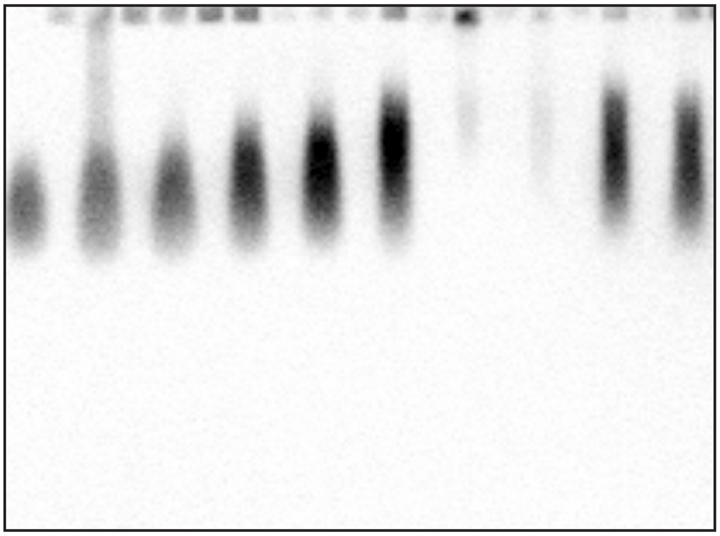

Figure S2

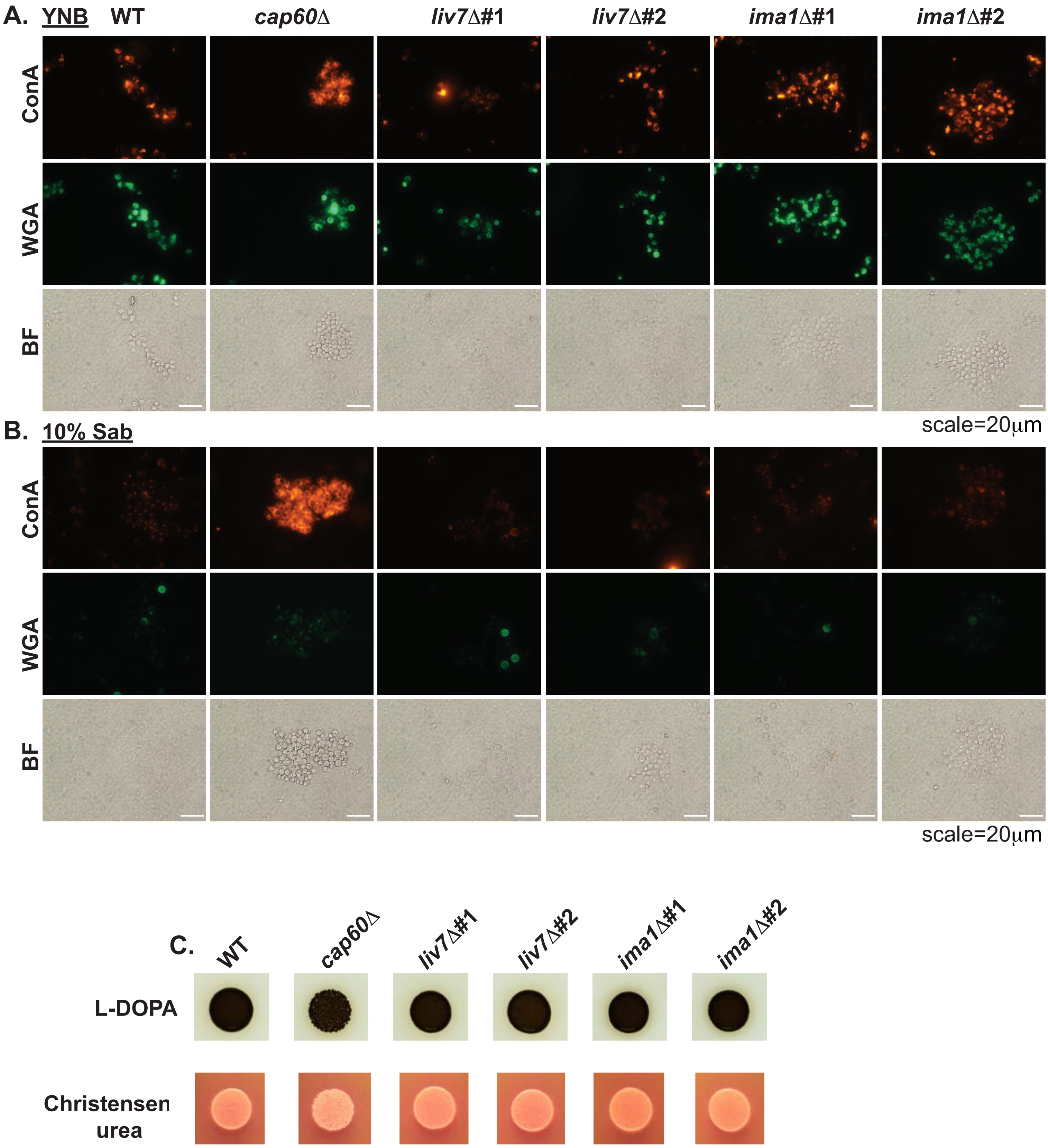
A.
YNB
WT
cap60Δ
liv7Δ#1
liv7Δ#2
ima1Δ#1
ima1Δ#2
ConA
WGA
BF
scale=20μm
B.
10% Sab
ConA
WGA
BF
scale=20μm
C.
WT
cap60Δ
liv7Δ#1
liv7Δ#2
ima1Δ#1
ima1Δ#2
L-DOPA
Christensen urea

Figure S3

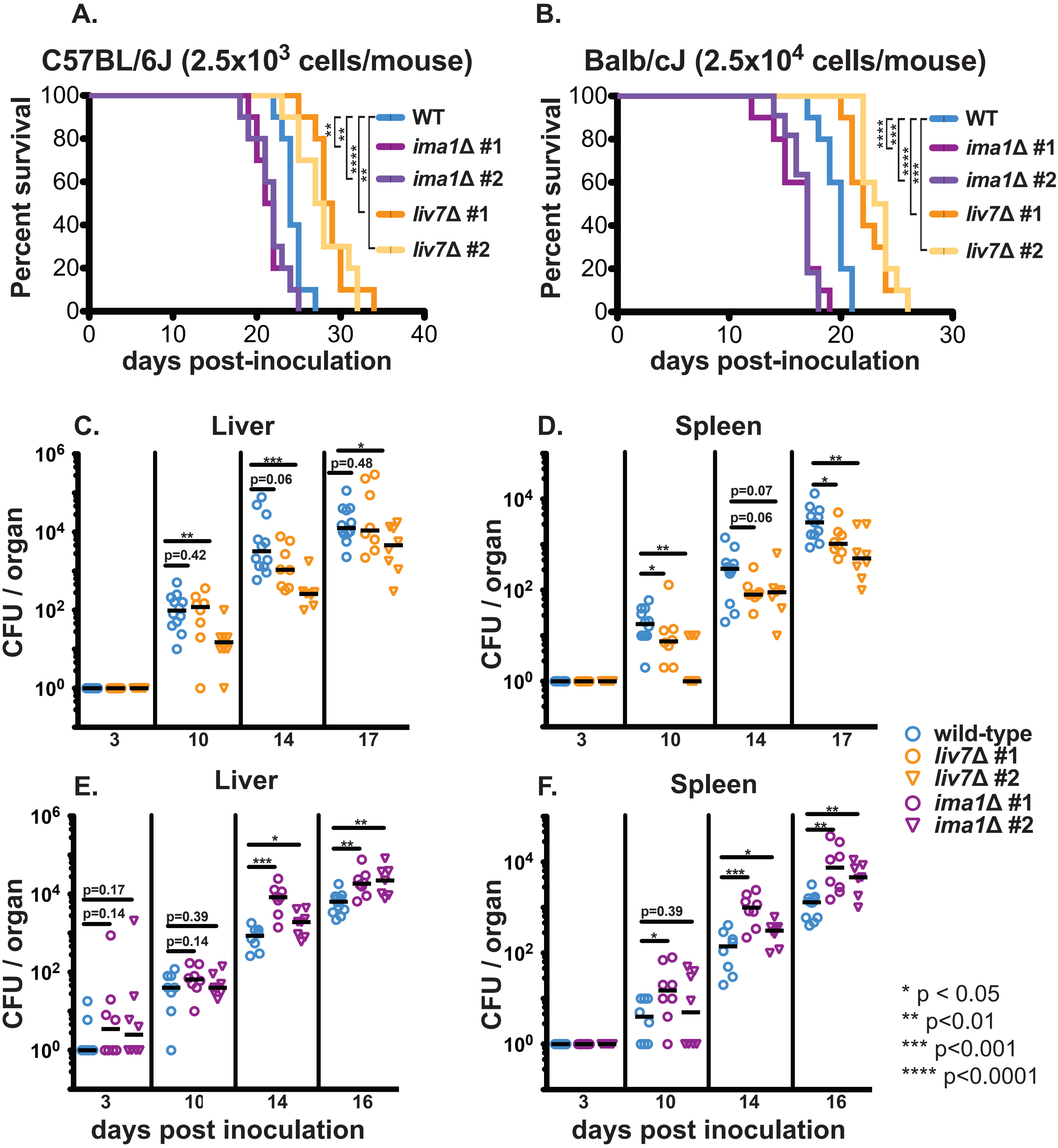

A.
C57BL/6J (2.5x10^3 cells/mouse)
Percent survival
days post-inoculation
WT
ima1Δ #1
ima1Δ #2
liv7Δ #1
liv7Δ #2
B.
Balb/cJ (2.5x10^4 cells/mouse)
Percent survival
days post-inoculation
C.
Liver
CFU / organ
D.
Spleen
CFU / organ
wild-type
liv7Δ #1
liv7Δ #2
ima1Δ #1
ima1Δ #2
E.
Liver
CFU / organ
days post inoculation
F.
Spleen
CFU / organ
days post inoculation
* p < 0.05
** p<0.01
*** p<0.001
**** p<0.0001

Figure S4

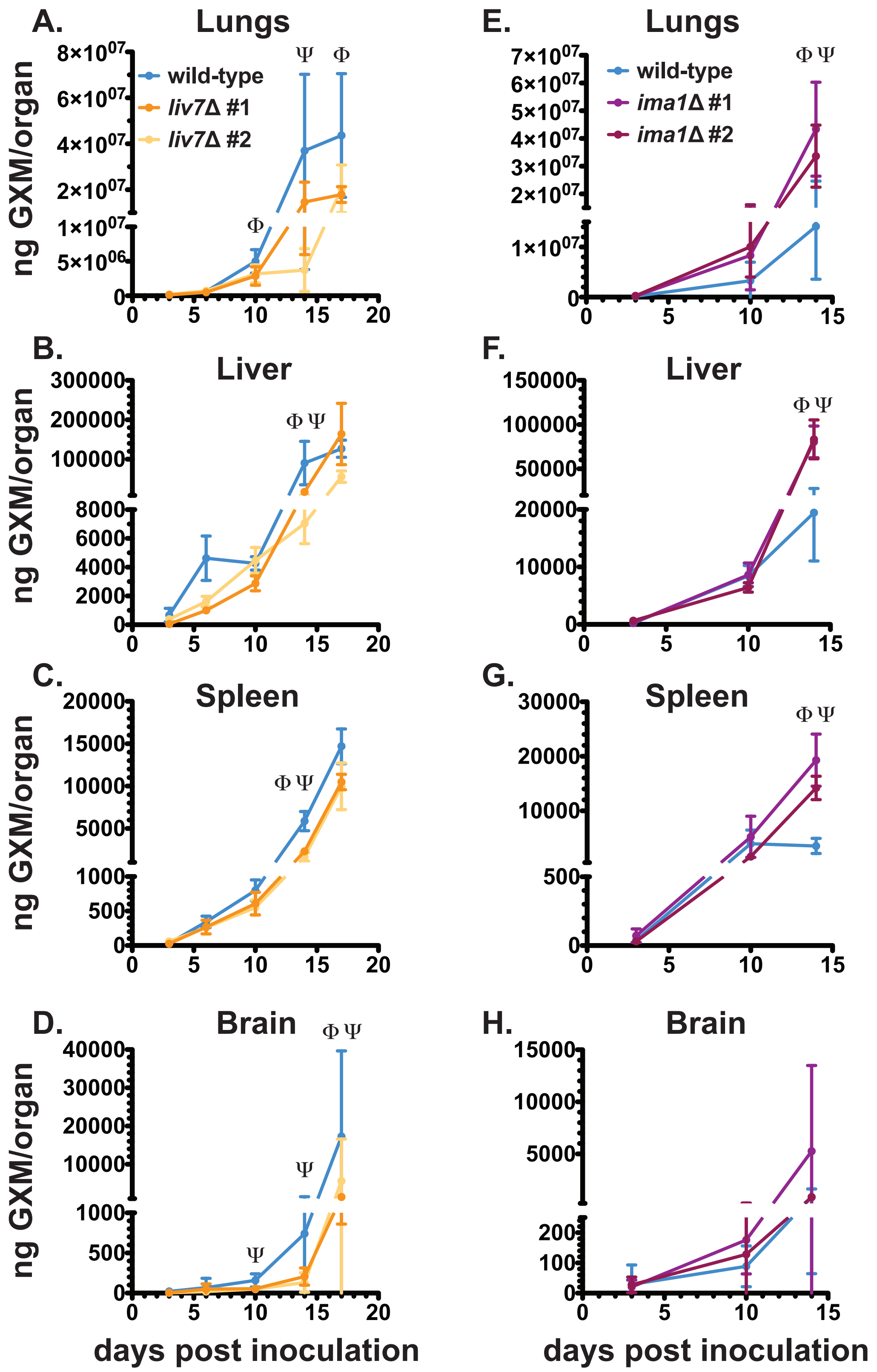

Φ $p<0.05$ (wild-type versus independent ko #1)

Ψ $p<0.05$ (wild-type versus independent ko #2)

Figure S5

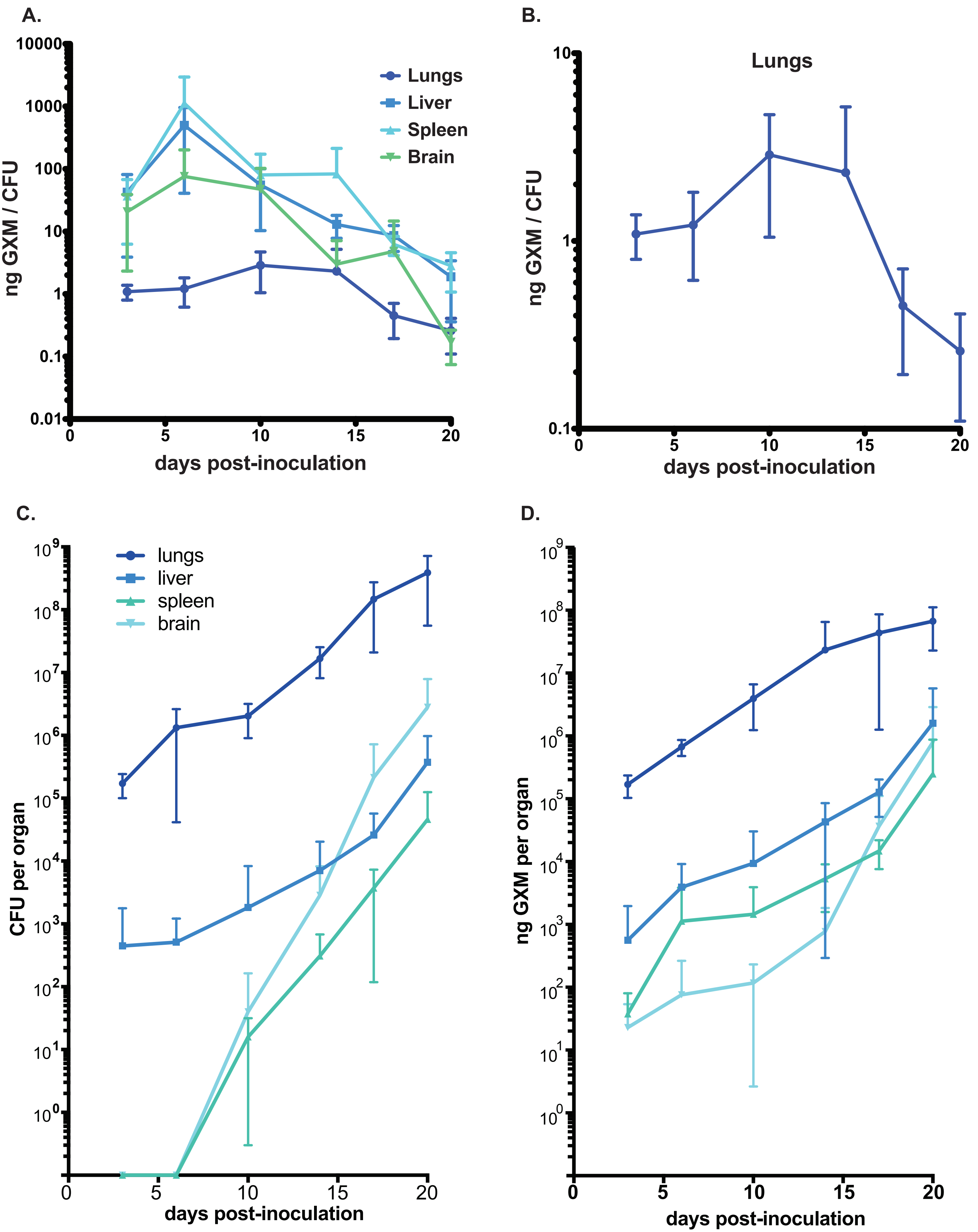

A.
ng GXM / CFU
10000
1000
100
10
1
0.1
0.01
0
5
10
15
20
days post-inoculation
Lungs
Liver
Spleen
Brain
B.
Lungs
ng GXM / CFU
10
1
0.1
0
5
10
15
20
days post-inoculation
C.
CFU per organ
lungs
liver
spleen
brain
0
5
10
15
20
days post-inoculation
D.
ng GXM per organ
0
5
10
15
20
days post-inoculation

Figure S6

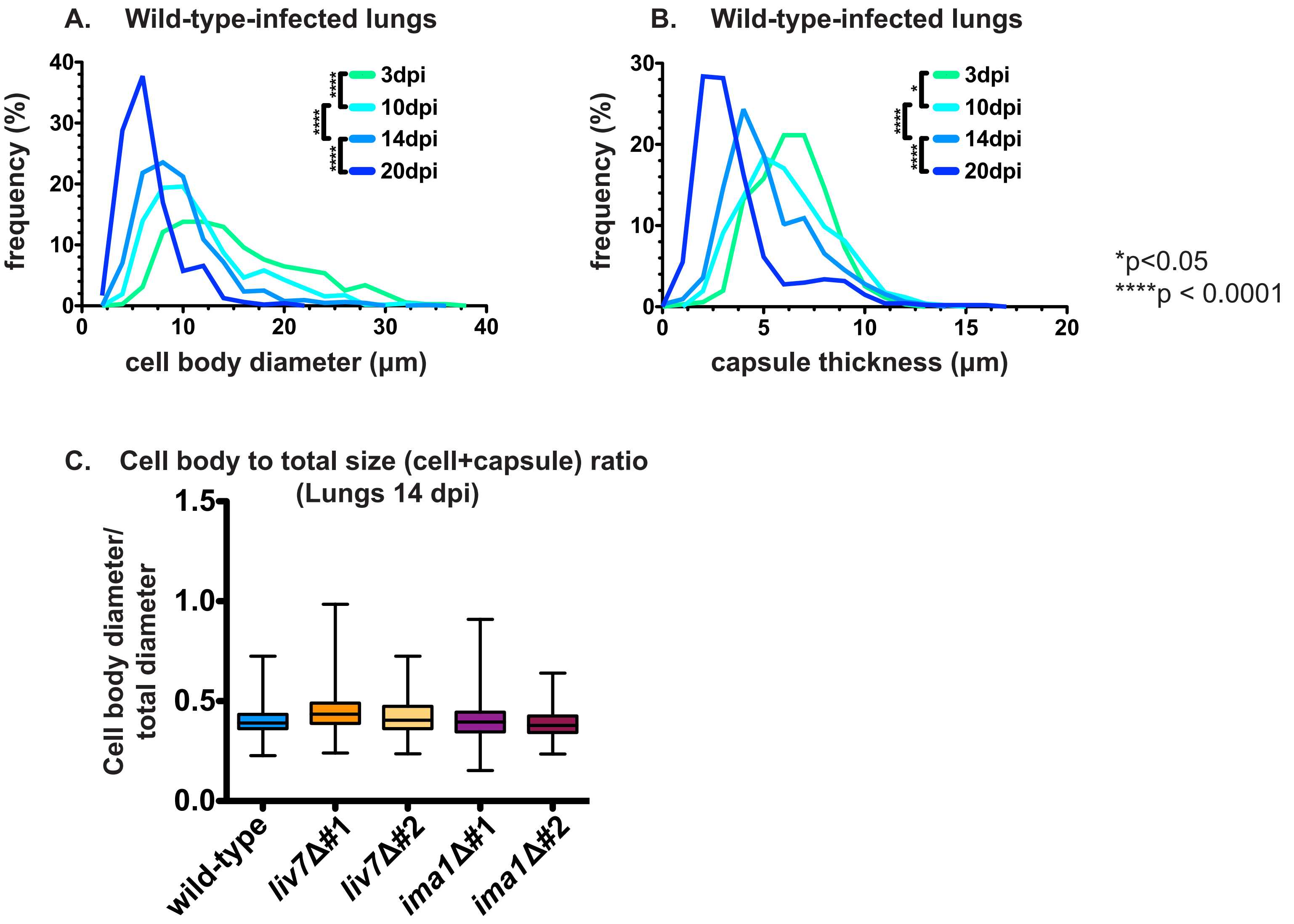

A. Wild-type-infected lungs
frequency (%)
cell body diameter (µm)
3dpi
10dpi
14dpi
20dpi
****
B. Wild-type-infected lungs
frequency (%)
capsule thickness (µm)
*
C. Cell body to total size (cell+capsule) ratio (Lungs 14 dpi)
Cell body diameter/ total diameter
wild-type
liv7Δ#1
liv7Δ#2
ima1Δ#1
ima1Δ#2
*p<0.05
****p < 0.0001

Figure S7

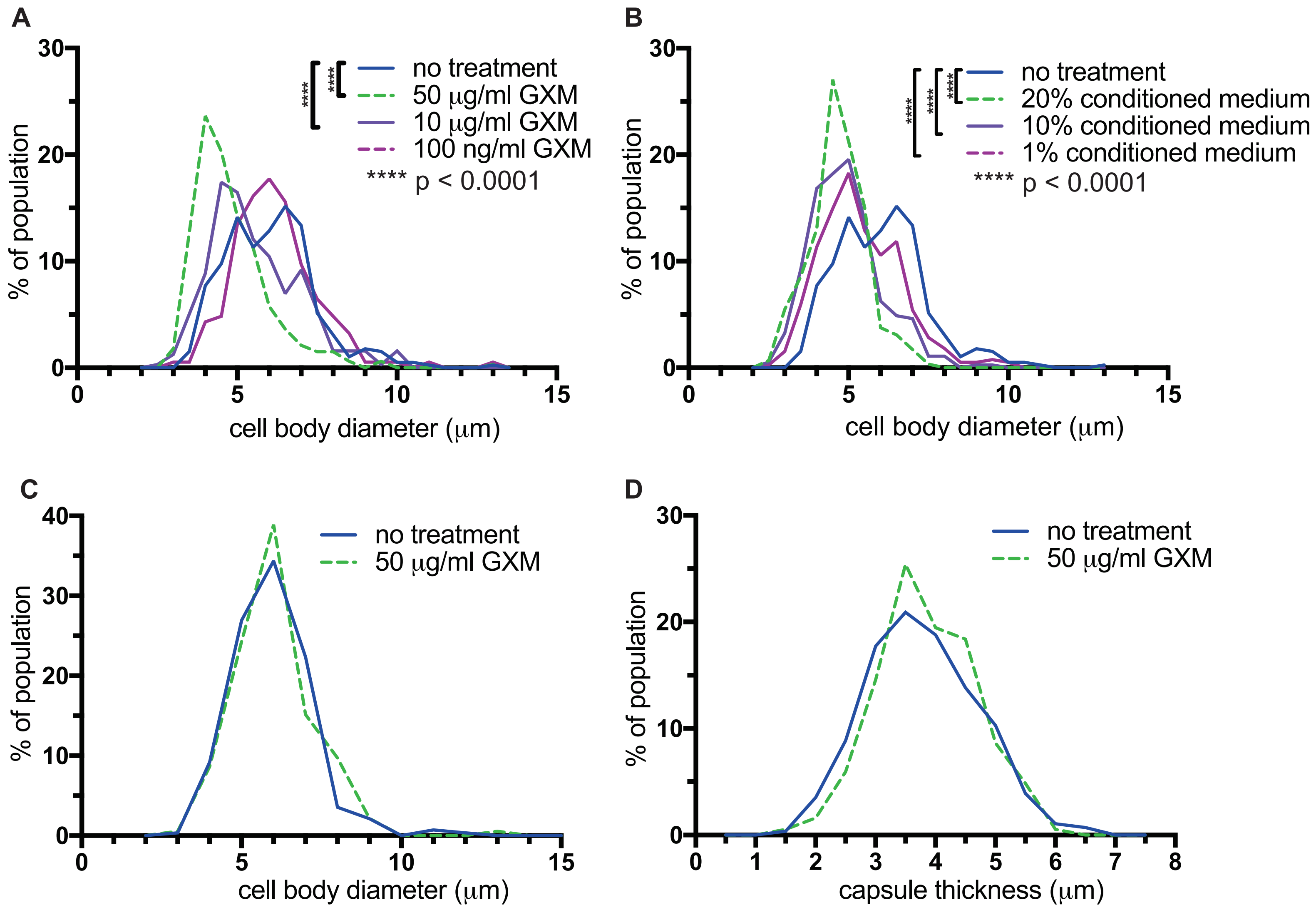
A
30
20
10
0
% of population
no treatment
50 μg/ml GXM
10 μg/ml GXM
100 ng/ml GXM
**** p < 0.0001
****
****
0
5
10
15
cell body diameter (μm)
B
no treatment
20% conditioned medium
10% conditioned medium
1% conditioned medium
**** p < 0.0001
cell body diameter (μm)
C
40
no treatment
50 μg/ml GXM
cell body diameter (μm)
D
no treatment
50 μg/ml GXM
capsule thickness (μm)

Figure S8

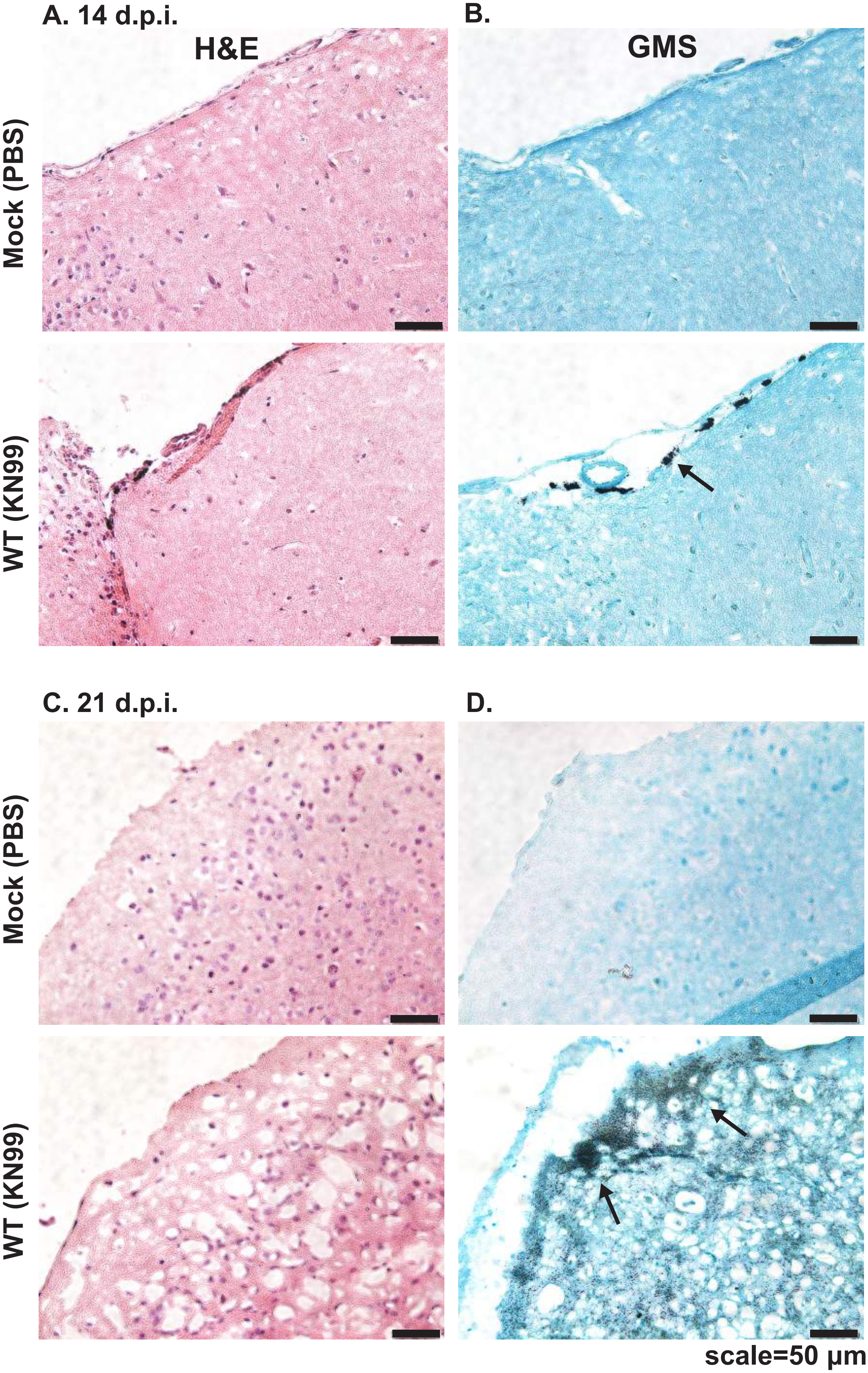

A. 14 d.p.i.
B.
H&E
GMS
Mock (PBS)
WT (KN99)
C. 21 d.p.i.
D.
Mock (PBS)
WT (KN99)
scale=50 µm

Figure S9

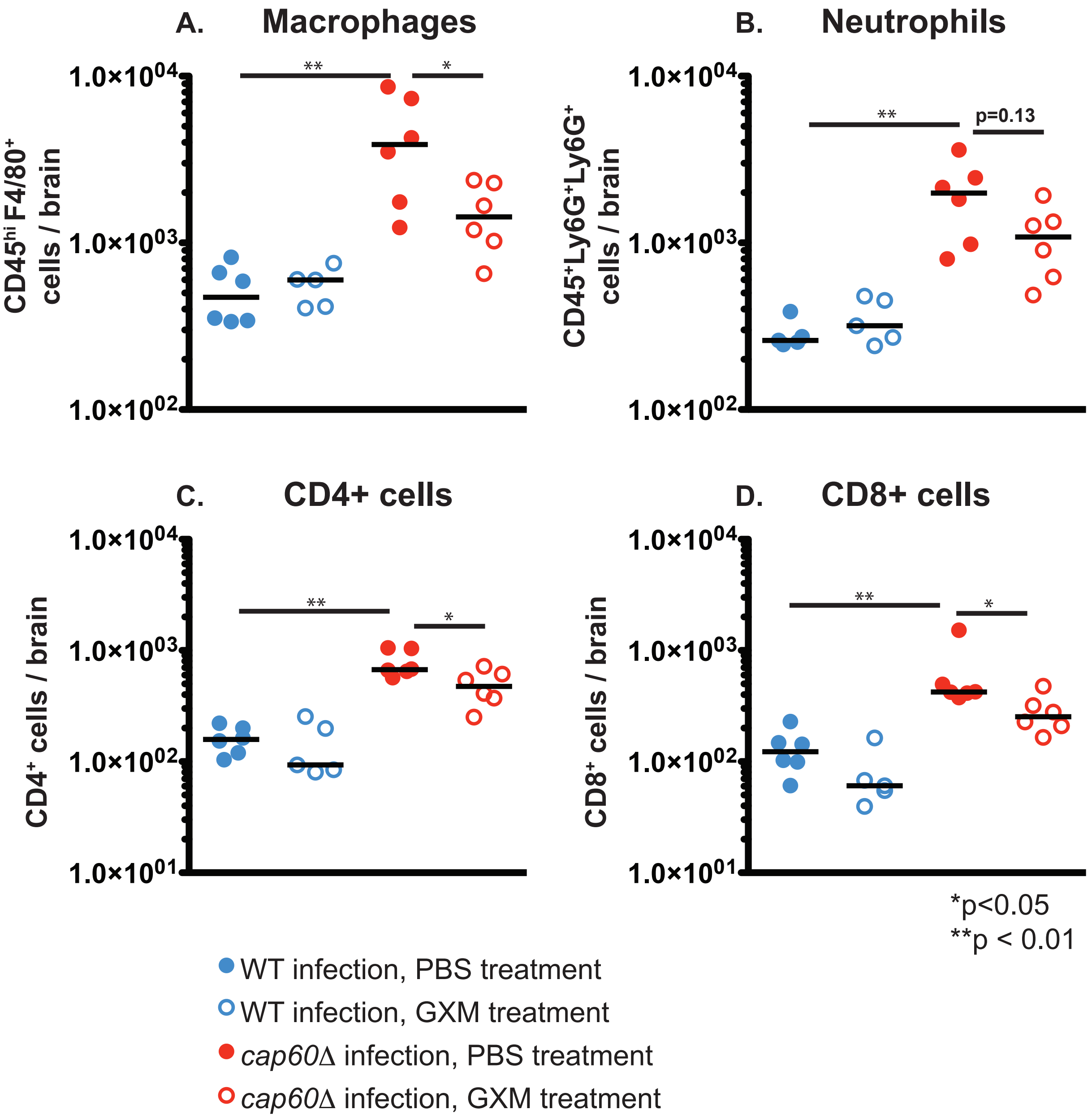

A.
Macrophages
CD45hi F4/80+ cells / brain
1.0×1004
1.0×1003
1.0×1002
**
*
B.
Neutrophils
CD45+Ly6G+Ly6G+ cells / brain
1.0×1004
1.0×1003
1.0×1002
**
p=0.13
C.
CD4+ cells
CD4+ cells / brain
1.0×1004
1.0×1003
1.0×1002
1.0×1001
**
*
D.
CD8+ cells
CD8+ cells / brain
1.0×1004
1.0×1003
1.0×1002
1.0×1001
**
*
*p<0.05
**p < 0.01
WT infection, PBS treatment
WT infection, GXM treatment
cap60Δ infection, PBS treatment
cap60Δ infection, GXM treatment
